# Epigenetic predictors of species maximum life span and other life-history traits in mammals

**DOI:** 10.1126/sciadv.adm7273

**Published:** 2024-06-07

**Authors:** Caesar Z. Li, Amin Haghani, Qi Yan, Ake T. Lu, Joshua Zhang, Zhe Fei, Jason Ernst, X. William Yang, Vadim N. Gladyshev, Todd R. Robeck, Andreas S. Chavez, Joseph A. Cook, Jonathan L. Dunnum, Ken Raj, Andrei Seluanov, Vera Gorbunova, Steve Horvath

**Affiliations:** ^1^ Department of Biostatistics, Fielding School of Public Health, University of California, Los Angeles, Los Angeles, CA, USA.; ^2^ Johnson & Johnson Innovative Medicine, Spring House, PA, USA.; ^3^ Department of Human Genetics, David Geffen School of Medicine, University of California, Los Angeles, Los Angeles, CA, USA.; ^4^ Altos Labs, San Diego, CA, USA.; ^5^ Department of Neurology, David Geffen School of Medicine, University of California, Los Angeles, Los Angeles, CA, USA.; ^6^ Department of Statistics, University of California, Riverside, Riverside, CA, USA.; ^7^ Department of Biological Chemistry, University of California, Los Angeles, Los Angeles, CA, USA.; ^8^ Center for Neurobehavioral Genetics, Semel Institute for Neuroscience and Human Behavior, University of California, Los Angeles, Los Angeles, CA, USA.; ^9^ Department of Psychiatry and Biobehavioral Sciences, David Geffen School of Medicine, University of California, Los Angeles, Los Angeles, CA, USA.; ^10^ Division of Genetics, Department of Medicine, Brigham and Women’s Hospital, Harvard Medical School, Boston, MA, USA.; ^11^ Zoological Operations, SeaWorld Parks and Entertainment Inc., Orlando, FL, USA.; ^12^ Department of Evolution, Ecology and Organismal Biology, The Ohio State University, Columbus, OH, USA.; ^13^ Translational Data Analytics Institute, The Ohio State University, Columbus, OH, USA.; ^14^ Department of Biology and Museum of Southwestern Biology, University of New Mexico, Albuquerque, NM, USA.; ^15^ Altos Labs, Cambridge, UK.; ^16^ Departments of Biology and Medicine, University of Rochester, Rochester, NY, USA.

## Abstract

By analyzing 15,000 samples from 348 mammalian species, we derive DNA methylation (DNAm) predictors of maximum life span (*R *= 0.89), gestation time (*R* = 0.96), and age at sexual maturity (*R *= 0.85). Our maximum life-span predictor indicates a potential innate longevity advantage for females over males in 17 mammalian species including humans. The DNAm maximum life-span predictions are not affected by caloric restriction or partial reprogramming. Genetic disruptions in the somatotropic axis such as growth hormone receptors have an impact on DNAm maximum life span only in select tissues. Cancer mortality rates show no correlation with our epigenetic estimates of life-history traits. The DNAm maximum life-span predictor does not detect variation in life span between individuals of the same species, such as between the breeds of dogs. Maximum life span is determined in part by an epigenetic signature that is an intrinsic species property and is distinct from the signatures that relate to individual mortality risk.

## Teaser

DNA methylation can estimate maximum mammal life span, but the predictor does not correlate with individual mortality risk.

## INTRODUCTION

Maximum life span varies markedly across mammalian species: The cinereus shrew lives less than 1.9 years, while bowhead whales can live for at least 211 years (*1*). The species appear to exhibit a maximum life span—an intrinsic characteristic of a biological species defined as the longest time an individual of a species has been reported to survive. However, the molecular mechanisms that determine it remain poorly understood (*2*, *3*), despite previous studies correlating maximum life span with specific molecular processes and life-history strategies (*4*–*6*). Many have suggested that epigenetic mechanisms play a role in determining life span (*7*–*15*). However, previous studies of cross-species variation in methylation patterns suffer from low sample size and heterogeneity in data acquisition methods.

To facilitate rigorous methylation studies of life-history traits, the Mammalian Methylation Consortium generated an unprecedented and homogeneous dataset of DNA methylation (DNAm) at well conserved loci across 348 mammals using a tailor-made DNAm measurement platform (*16*). Other reports by the Consortium have described pan-mammalian age-related methylation changes, epigenetic aging clocks, phylo-epigenetic trees, and unsupervised machine learning approaches that were brought to bear on the analyses of this dataset (*17*, *18*). In recent publications by the Mammalian Methylation Consortium, we released a DNAm dataset (*n* = 15,456 tissue samples) (*17*, *18*). These previous investigations uncovered individual cytosines and modules that correlate with maximum life span, gestation time, and age at sexual maturity.

Here, we pivot our analytical approach. Rather than seeking individual cytosine-phosphate-guanines (CpGs) tied to maximum life span and other life-history traits, we develop regularized multivariate regression models that estimate maximum life span and other characteristic traits of species. Drawing on statistical terminology, our previous work focused on univariate analysis (specifically, the selection of CpGs) and CpG modules (*18*). In contrast, here we use multivariate regression models to predict maximum life span (the dependent variable), based on highly conserved cytosines (the independent variables or covariates) simultaneously. Using this approach, we successfully developed methylation-based predictors of time-related life-history traits: maximum life span, gestation time, and age at sexual maturity across mammalian species. Next, we characterized these new epigenetic biomarkers with regard to a variety of conditions ranging from demographic characteristics (age, sex, human mortality risk) to interventions that modulate murine life span.

## RESULTS

### DNAm data from 348 mammalian species

Leveraging our publicly accessible data from the Mammalian Methylation Consortium, we focused on highly conserved cytosine methylation profiles from *n* = 15,000 DNA samples (*18*). These samples spanned 59 unique tissue types and originated from 348 distinct mammalian species across 25 taxonomic orders. In total, the Mammalian Consortium profiled 25 of the 26 mammalian taxonomic orders as catalogued in the Mammal Diversity Database (version 1.8, 2022), with marsupial moles being the only exception.

These methylation profiles were obtained using the mammalian methylation array, a tailor-made DNA array developed for the consortium’s objectives (*16*). This array efficiently gauges the methylation levels of roughly 36,000 highly conserved CpG sites. These CpGs are flanked by 50–base pair DNA sequences that are remarkably conserved across various mammalian species.

### Universal predictors of sex and tissue type

The mammalian array–generated DNAm data prove highly effective in accurately classifying sample species, sex, and tissue. This is supported by our random forest predictors, which boast an out-of-bag accuracy rate of over 98% (Table 1). We present universal sex predictors grounded in CpG methylation levels that are applicable to all mammalian species, barring marmosets (Table 1). It is widely recognized that mosaicism in marmosets hinders the creation of methylation-based sex predictors for them (*19*). We previously postulated that the inability to build methylation-based predictors of sex in marmosets is due to their nature as hematopoietic chimeras. Specifically, littermates in marmosets exchange stem cells through placental anastomoses during development, as discussed in (*19*).

**Table 1.** Sex and pan-tissue predictor performance. The table summarizes test set prediction results for regularized regression-based predictors and out-of-bag prediction results for random forest (RF)–based predictors. For the sex elastic net linear predictor, test sets are randomly partitioned into equal 10 folds of the entire dataset. At each iteration, within the 90% training set, 10-fold validation was used to select the penalization parameter for the regularized regression. For random forest predictors, we specified 100 trees in the forest. To counteract the imbalance in tissue and species category sample sizes, we limited the bootstrap resampling to 200 per category, restricting large sample sizes from certain categories, such as blood samples, overly influencing the predictor performance in smaller categories.

Predicted outcome	Predictor framework	Method	Test set/out-of-bag prediction accuracy
Sex (female = yes/no)	Elastic net	10-fold validation	98.53%
Species	RF predictor	*100 trees	99.94%
Tissue	RF predictor	100 trees	98.22%
Taxonomic order	RF predictor	100 trees	99.97%

*100: each random forest was calibrated to use this many decision trees with a reasonable run time; random forest unbiased prediction accuracy estimate is calculated as follows: first, summarize by calculating the mean of each category’s out-of-bag prediction errors, subtracted by unity, across the 100 trees; second, use these category mean accuracies to find the overall median-of-means accuracy. Marmosets were removed from the sex prediction analysis.

Our universal tissue predictors, based on methylation, are likely influenced by species variations. While we offer these tissue predictors to the community as tools for identifying human platemap errors, we advise users to be aware of the potential species-related confounding factors associated with these predictors.

### Multivariate predictors of life-history traits

Since we aimed to focus on species traits, we first reduced the confounding effect of sex and tissue type by averaging across these variables. Specifically, we calculated the mean methylation value for each CpG within each species, producing a summarized dataset in which each data point corresponds to a species’ average methylation level per CpG (table S1). In addition to this overarching dataset, we curated two more specialized datasets: one stratified by both species and tissue type and another that exclusively focuses on younger samples that were derived from animals that are both not yet sexually mature and under 5 years of age.

**Fig. 1.**
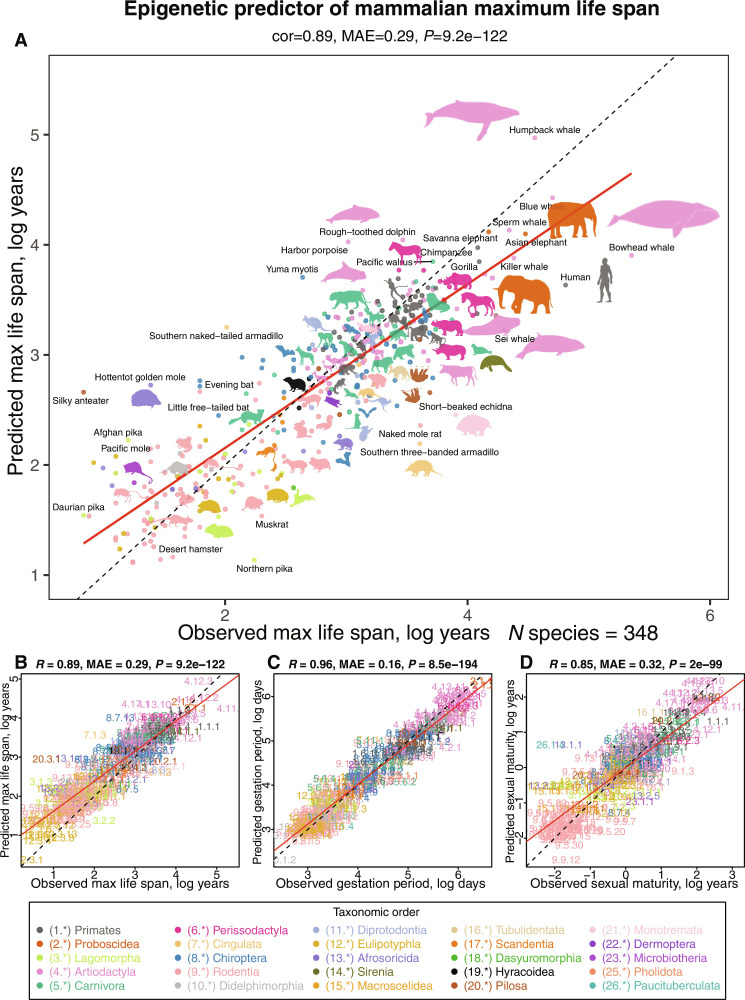
Multivariate analysis of life-history traits using epigenetic predictors. This figure summarizes the leave-one-species-out (LOSO) cross-validation analysis of epigenetic predictors. All estimates are log-transformed (base e) for various life-history traits, including (**A** and **B**) maximum life span (in log years), (**C**) gestation time (in log days), and (**D**) age at sexual maturity (in log years). Each species in the scatterplot panels is symbolized by a specific number. The whole number (integer) part of this numeric representation corresponds to its taxonomic order. These numbers, color-coded by their respective taxonomic orders, link to distinct species. For detailed numeric values, refer to table S4 and fig. S8. The title atop each panel provides Pearson correlation coefficient (*R*), median absolute error (MAE), and a two-sided unadjusted *P* value (*P*). Consistency in color representation for taxonomic orders is maintained throughout this and other related figures. A dotted line within the scatterplots represents the line of perfect prediction, while the solid red line is the fitted linear regression. Animal silhouettes featured are sourced from the Phylopic database (https://www.phylopic.org/) or Wikimedia, which are under public domains or the CC BY 3.0 license (https://creativecommons.org/licenses/by/3.0/).

**Fig. 2.**
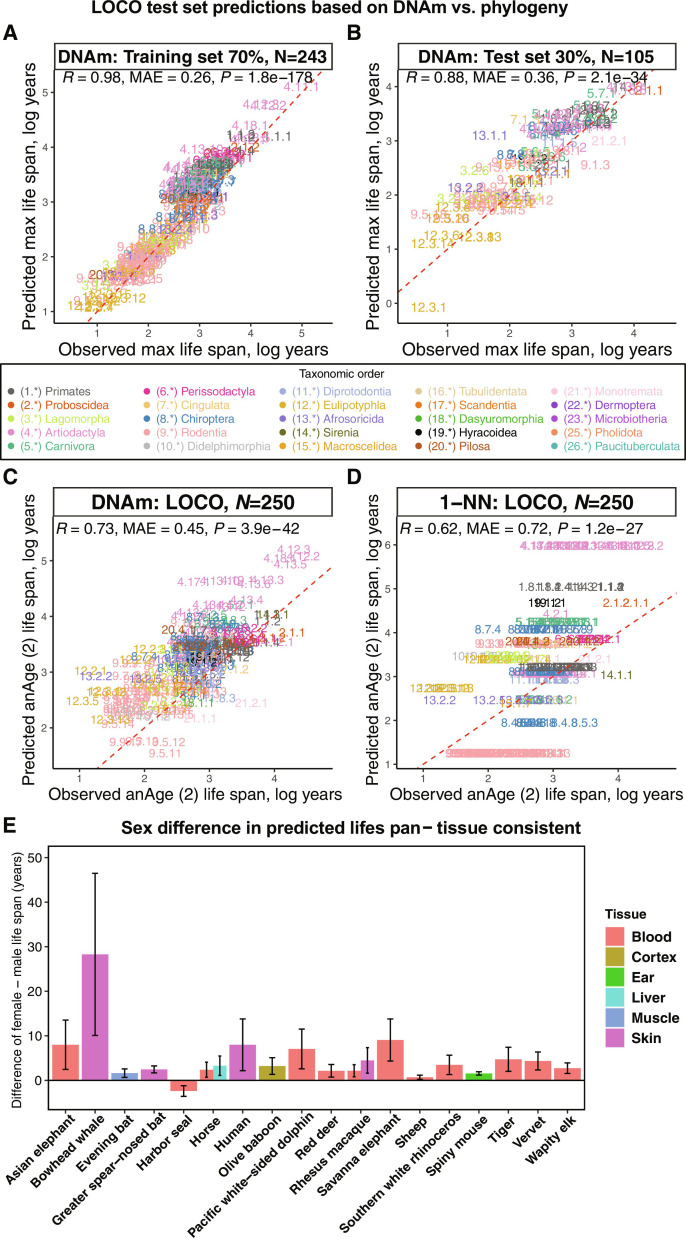
Comparison of DNAm life-span predictor, phylogeny-based predictor, and sex-related differences in predicted life span. (**A** and **B**) Evaluation of the multivariate predictor of maximum life span based on cytosine methylation in training data (A) and test data (B), encompassing 70% and 30% of species, respectively. In (A) and (B), each data point symbolizes a unique species, differentiated by its taxonomic order color coding. The dotted red line indicates the fitted linear regression. (**C** and **D**) Leave-one-clade-out (LOCO) cross-validation analyses concentrate on the log-transformed (base e) maximum life-span predictions. Given that several species’ missing life-span observations were filled using neighboring species, life-span estimates naturally favor k-NN. To mitigate this bias, this analysis only includes 250 species from the original anAge database (*2*) with actual maximum life-span records. This analysis provides an unbiased assessment of the performance of the DNAm elastic net predictors (C) compared to the k-nearest neighbor (k-NN with K = 1) predictor (D), which uses distances from the mammalian phylogenetic TimeTree (*55*). (**E**) Bar plot reports the differences in life-span predictions between females and males by tissues, specifically highlighting species that exhibits uniformity across tissues with statistically significant (two-sided unadjusted Wilcoxon rank sum test, *P* ≤ 0.01) female-male differences. This means that in all statistically significant tissue groups, females are consistently predicted to have longer DNAm life span. Error bars outline the 95% confidence interval (CI) of these differences. Bars throughout the figure are colored by tissue type, as detailed in the accompanying legend.

We used three distinct penalized regression models to predict the log-transformed values of maximum life span, gestation time, and age at sexual maturity for each species. The trait values for these species were derived from the latest version of the anAge database (*2*, *18*). For the convenience of our readers, we have included these values in tables S1 and S2. The resultant epigenetic predictors showcased high accuracy as evidenced by the leave-one-species-out (LOSO) or a modified leave-one-clade-out (LOCO) cross-validation. For instance, the predicted log maximum life spans aligned closely with those recorded in anAge, exhibiting a Pearson’s correlation of *R* = 0.89 (see Fig. 1, A and B). An alternative method for assessing predictive precision entails dividing the data into training and test subsets. Using our 70%-30% training-test random partitioning of species, we observed comparably robust correlations for the log maximum life span in both subsets (training set, *R* = 0.98, Fig. 2A; test set, *R* = 0.88, Fig. 2, A and B).

Shifting our focus to other life-history traits, the actual log gestation time—which is inherently more straightforward to determine than maximum life span—manifested an even higher correlation with its predicted counterpart (*R* = 0.96, Fig. 1C). The epigenetic prediction of (log-transformed) age at sexual maturity presented a somewhat lower correlation of *R* = 0.85 with recorded data (Fig. 1D). This discrepancy might stem from the fact that the age at sexual maturity is considerably more variable than gestation time, being influenced by factors like food availability and varying ecological conditions.

We will refer to the predicted maximum life span, expressed in log years, as either the epigenetic maximum life span or DNAm maximum life span. Analogous naming conventions will apply to other DNAm-derived estimates of life-history traits. The final life-history predictor coefficients, which were trained on all available samples, and the corresponding CpG annotations are summarized in tables S5 to S7 (also available on Zenodo, https://doi.org/10.5281/zenodo.10783145, supplemented by the R package on Github: https://github.com/caeseriousli/MammalianMethylationPredictors).

### Chronological age versus epigenetic maximum life span

We carried out two analyses to study the relationship between the life-history traits and chronological age of the individuals of species sampled. First, we built a separate maximum life-span predictor (referred to as young animal predictor) using only samples obtained from animals that were younger than their species’ average age of sexual maturity and younger than 5 years, and this had a considerable correlation with predicted maximum life span (*R* = 0.68, fig. S1), although the restriction of age resulted in fewer species (*n* = 122) being available for this analysis. The young animal predictor’s remarkable accuracy in long-lived species (for instance, those with a maximum life span exceeding 20 years) indicates that the determinants of maximum life span can be discerned from DNA samples obtained even from relatively young individuals.

Second, we used the finalized life-span predictor model on individual animal samples. While the predictor was designed to estimate species-level life span on a logarithmic scale, we used the coefficients to predict the life span of individual samples. Our findings indicate that the predicted maximum life spans for individual samples can vary and, in certain species such as the naked mole rat skin, human blood, sheep ear, and cat blood, correlate significantly (Pearson correlation test *P* < 0.05) with chronological age (fig. S2). In a similar vein, gestation duration and age of sexual maturity correlate significantly with age (*P *< 0.05) in select species-tissue strata (figs. S3 and S4). Overall, our analysis (figs. S2, S3, and S4) reveals that epigenetic indicators of life-history traits, when confined to a specific species and tissue, do not have a consistent correlation with age.

### Effect of tissue type

In the preceding section, we introduce epigenetic predictors for life-history traits, derived from mean methylation levels averaged across species and encompassing all available tissue types. As these predictors disregarded specific tissue types, we term these as tissue-agnostic life-history predictors.

To delve deeper into the influence of tissue type on life-span predictions, we applied these tissue agnostic epigenetic predictors—specifically for maximum life span, gestation duration, and age of sexual maturity—to selected species for which multiple tissue types were available (figs. S5 to S7).

The epigenetic maximum life-span estimates do reveal disparities between certain tissues. For instance, in human samples, distinct epigenetic life-span predictions emerge (table S3): Blood and epidermis yield elevated life-span predictions of 98.1 and 94.6 years, respectively, while skin and cerebral cortex produce estimates of 79.1 and 51.1 years, respectively. In contrast, embryonic stem cells (34.4 years), induced pluripotent stem cells (iPSCs) (25.6 years), endothelial cells (23.9 years), and skeletal muscle (35.4 years) present lower life-span predictions (table S3).

The trend of blood samples reflecting the highest epigenetic maximum life span is consistent across various species (fig. S5). For instance, in species ranging from humans to brown rats, blood samples consistently indicate elevated epigenetic maximum life-span predictions.

In horses, we have observed that blood results in elevated life-span predictions, whereas the ovaries and adrenal cortex yield lower estimates (fig. S5). In mice, blood, Lin(−)Sca-1(+)c-Kit(−) [LSK(−); which are cell surface markers to characterize a subset of cells within the bone marrow that is capable of self-renewal and differentiation into all types of blood cells] progenitor hematopoietic stem cells, and bone marrow macrophages stand out with elevated predictions, whereas other tissues align closely. Both beluga whales and rhesus macaques show elevated life-span estimates in blood (fig. S5). In summary, blood samples consistently yield higher epigenetic maximum life-span predictions across a variety of species. A detailed overview is available in table S3. The biological significance of these disparities warrants further investigation. While the preceding analysis focused on maximum life span, we also conducted evaluations of tissue disparities in the predicted gestation time (fig. S6) and the age at which sexual maturity is reached (fig. S7).

We briefly describe a strategy for building epigenetic predictors of life-history traits that mitigate the confounding influence of tissue types. A predictor for maximum life span can be built based on mean methylation levels in strata formed by species and tissue type, termed tissue-aware life-history predictors. In this setup, every species is represented through multiple data points corresponding to different tissues collected from the same species. Notably, these predictors, rooted in species-tissue aggregated data, are highly accurate (fig. S8). In addition, maximum life span, gestation time, and age at sexual maturity predictors produce similar tissue-stratified results (figs. S9 to S11). The predictor coefficients and corresponding CpG annotations are summarized in tables S8 to S10 (also available on Zenodo, https://doi.org/10.5281/zenodo.10783145, supplemented by the R package on Github: https://github.com/caeseriousli/MammalianMethylationPredictors). In our subsequent discussions and the remainder of the article, we will focus on tissue-agnostic predictors for life-history traits.

### Superior performance of DNAm-based predictors

While DNAm levels are influenced by genetics, our DNAm-based life-span predictor seems to transcend DNA sequence variation influenced by phylogenetic relationships. This assertion is supported by two distinct analyses.

First, we used elastic net regression models to predict maximum life span using both CpG methylation data and taxonomic order indicators. The model exclusively selected CpGs, indicating their superior explanatory power over taxonomic variables in life-span variation.

Second, we compared the accuracy of the epigenetic life-span predictor against k-nearest neighbor (k-NN) regression models, which base predictions on phylogenetic tree branch lengths. At its simplest, with *K* = 1, the k-NN model predicts a species’ life span based on its closest taxonomic neighbor. Upon evaluating the correlation between predicted and actual values, the phylogeny-driven k-NN model slightly trails the DNAm predictor, especially under a LOSO evaluation. This is primarily because many mammalian species in our dataset exhibit life spans akin to their taxonomic neighbors (fig. S12, A and B, and table S1). This trend is also pronounced at the taxonomic family level (fig. S12, C and D). However, the k-NN model’s performance diminishes under a more rigorous LOCO evaluation, which tests the model’s ability to predict life span of taxonomically diverse species. While k-NN models (with *K* = 1) achieved a moderate correlation of *R* = 0.62 (Fig. 2D and fig. S13), they lag behind the methylation-based predictor, which boasts a correlation of *R* = 0.73 (Fig. 2C and fig. S12). k-NN models with *K* = 2 and *K* = 3 neighbors yielded correlations of *R* = 0.62 and *R* = 0.57, respectively. A detailed examination of the residuals highlights the k-NN model’s tendency to make generalized predictions for larger taxonomic orders, often deviating notably from actual values (fig. S13).

In conclusion, when assessed through LOCO cross-validation, DNAm-based predictors improve upon their phylogeny-based counterparts. The DNAm predictor’s capability to accurately estimate life span across diverse taxonomic orders underscores its potential to capture aspects of mammalian life span that transcend phylogenetic relationships.

### Sex differences in predicted life span

We aimed to investigate any potential disparities in maximum life-span predictions across sexes. Using our final regression model, based on average methylation data per species and designed to predict species-level life span on a logarithmic scale, we predicted individual sample life spans. Predictions from female tissues showed a striking alignment with those from male tissues, with a strong correlation of *R* = 0.99 on a log scale. Most species showed consistent epigenetic estimates of maximum life span in female and male samples (tables S3 and S4, column “Female–Male Significant Tissues,” where “+” denotes female minus male mean predicted DNAm life span is positive with an unadjusted *P* value of ≤0.01, “-” vice versa, and “.” denotes a *P* value of >0.01). Stratifying by tissue type, we observed statistically significant consistent sex difference in epigenetic maximum life span (a more conservative two-sided unadjusted Wilcoxon rank sum test, *P* < 0.01) (*20*) in only 18 species (Fig. 2E). This means that we only consider it statistically significant when (i) at least one tissue group within the species exhibits statistically significant (two-sided unadjusted Wilcoxon rank sum test, *P* ≤ 0.01) female-male differences and (ii) these female-male differences must be in the same direction, for example, the female mean DNAm life span being consistently greater than that of the males. In other words, we look for species for which one sex is consistently predicted to have longer DNAm life span than the other. Females were predicted to have a longer maximum life span than males in 17 of the 18 species, including humans (Fig. 2E and table S3). The one exception was blood from harbor seals. In a comparison across species, females exhibit an average of 1% longer predicted epigenetic maximum life span than their male counterparts, with an SD of 11.9% (details in table S3).

### Minor effect of adult weight in prediction accuracy

Across species, there is a notable positive correlation between maximum life span and average adult weight (body mass), as depicted in fig. S14A. This correlation has been well documented in previous studies (*2*). Given this, we evaluated whether the high accuracy of epigenetic life-span predictors could be influenced by the average adult weight. Our findings from two distinct analyses suggest otherwise.

In the first analysis, we focused on small animals, specifically those with an average adult weight of less than 150 g. Despite a negative correlation between adult weight and maximum life span in these species (*R* = −0.29, fig. S14C), the epigenetic predictor of maximum life span still showed a strong correlation with observed values (*R* = 0.53, *P* = 1.6 × 10^–10^, fig. S14B). In the second analysis, encompassing all animals, a multivariate regression model (with the dependent variable being the log of maximum life span) indicated that (log-transformed) adult weight (Wald test, *P* = 1.3 × 10^–6^) is a less significant covariate than (log-transformed) epigenetic maximum life span (*P* < 2 × 10^–16^). This shows that adult weight only weakly mediates the effect of epigenetic maximum life span on actual maximum life span. This observation is reinforced by a correlation value (*R*) of 0.54 between our model’s predictions, after weight adjustments, and the actual maximum life span (fig. S14D). In conclusion, both analyses consistently show that the epigenetic maximum life span provides predictive information that extends beyond adult weight.

### Cancer mortality risk across mammals

Distinct variations in cancer mortality rates across major mammalian orders have been documented (*21*). Notably, there exists a pronounced negative correlation between mammalian cancer risk and observed gestation time (Pearson *r* = −0.37, *P* = 0.0031, fig. S15). Considering the notable correlation among gestation time, maximum life span, and age at sexual maturity on a logarithmic scale (fig. S15, A and B), one might theorize that one or more of these life-history traits could predict cancer mortality risk in mammals. However, this theory is challenged by the data: Neither maximum life span (fig. S15D) nor average age at sexual maturity (fig. S15E) exhibits this anticipated relationship. The only significant correlation with cancer mortality risk is observed for gestation time and its epigenetic counterpart (*r* = −0.41, *P* = 0.00092, fig. S15I). Further, upon adjusting for observed life history traits, no significant correlation was found between epigenetic predictions of life-history traits and mammalian cancer risk (fig. S15, K to M). Collectively, these findings indicate that the epigenetic markers predicting life-history traits, such as gestation time, do not inherently offer predictive information into mammalian cancer risk beyond the observed life-history values. This result is consistent with the concept of Peto’s paradox where there is no correlation between cancer rates and either maximum life span or body mass (*21*).

### Weak effect of mutations in the somatotropic axis

The somatotropic axis, encompassing growth hormone, insulin-like growth factor–1 (IGF-1) levels, and their respective receptors, is a focal point in aging and longevity research (*22*). Growth hormone receptor knockout (GHRKO) mice (dwarf mice) typically exhibit an extended maximum life span (*23*, *24*). A full-body GHRKO mouse holds the record of nearly reaching a life span of 5 years (*22*). In our study, we sought to determine if decreased GH/IGF-1 pathway activity influences the epigenetic estimates of maximum life span across three distinct mouse models. It should be noted that Snell dwarf mice and full-body GHRKO mice show extended maximum life spans (*25*–*27*). On the other hand, liver-specific GHRKO mice, despite exhibiting reduced serum IGF-1 levels, do not show a corresponding increase in maximum life span (*28*, *29*).

**Fig. 3.**
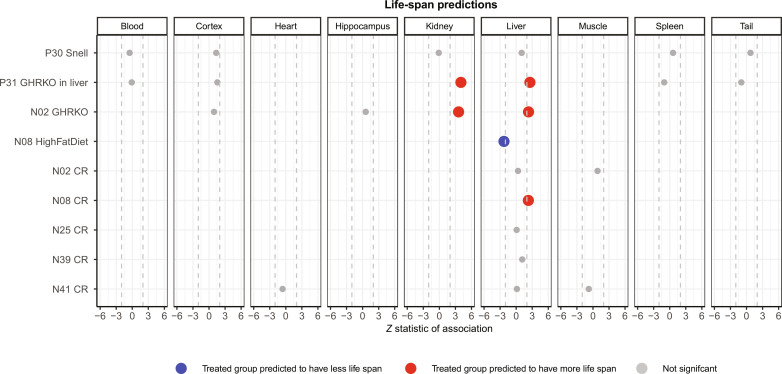
Predicted life span across murine experimental treatment groups. Predicted life span from the final model fitted to individual samples in murine perturbation experiments. Each row corresponds to a specific experiment, and columns stratify these results by tissue types. The experimental treatment groups, from top to bottom, are as follows: Snell dwarf mice, liver-specific growth hormone receptor knockout (GHRKO) mice, full-body growth hormone knockout mice, high-fat diet, and five separate caloric restriction (CR) experiments. The prefixes in the rows, such as P30 for “project 30” and N08 for “number 8,” denote distinct datasets. Empty cells denote the absence of samples for the corresponding tissue in the experiment. Gray dots represent associations that are not statistically significant. Red and blue markers highlight significant associations (*P* < 0.05) that align with our expectations. We found no significant associations that deviated from our expectations. The *x* axis reports Wald test statistics that follow a standard normal distribution under the null hypothesis. Dashed lines represent the critical *Z* statistic values when assessing a two-sided *t* test with type I error controlled at α = 0.05.

Our observations indicate that both the full-body GHRKO and liver-specific dwarf mice show a notably extended epigenetic maximum life span, particularly in samples from liver and kidney (Fig. 3). However, such association was not observed in samples from blood, cerebral cortex, hippocampus, spleen, or tail. Similarly, we did not detect any significant association across tissues in Snell dwarf mice. Given these observations, two potential inferences emerge. Either manipulation within the somatotropic axis (comprising growth hormone, IGF-1 levels, and their associated receptors) has at best a weak effect on epigenetic life-span estimators in select tissues, or the epigenetic predictor of maximum life span is insufficiently precise when used in mouse studies.

### Equivocal effect of caloric restriction and high-fat diet on epigenetic life span

Caloric restriction has been documented to extend the maximum life span in approximately one-third of all mouse strains. We aimed to gauge the influence of caloric restriction on the epigenetic estimates of maximum life span from mouse liver samples. In four of the five studies, no significant (when assessed with a relaxed, unadjusted type I error rate control of 5%) impact on epigenetic maximum life span in murine liver was observed (Fig. 3). Only one study presented the expected association between caloric restriction and a prolonged maximum life span (Fig. 3).

On the other hand, high-fat diets have been identified as factors that both shorten murine life span and accelerate epigenetic aging (*30*). Consistent with this, our observations did confirm the anticipated link between a high-fat diet and a reduction in epigenetic maximum life span (Fig. 3). In sum, the outcomes from the application of epigenetic maximum life-span indicators to mouse interventions, which inherently influence mouse longevity, are somewhat equivocal.

### Cellular reprogramming based on the Yamanaka factors

The Yamanaka factors, comprising *Oct4*, *Sox2*, *Klf4*, and *Myc* (OSKM), are known for their role in full reprogramming (resulting in iPSCs) as well as in partial reprogramming of somatic cells (*31*–*36*). We tested whether reprogramming affects epigenetic maximum life span using publicly accessible data from both complete and partial reprogramming studies conducted on human and mouse cells.

**Fig. 4.**
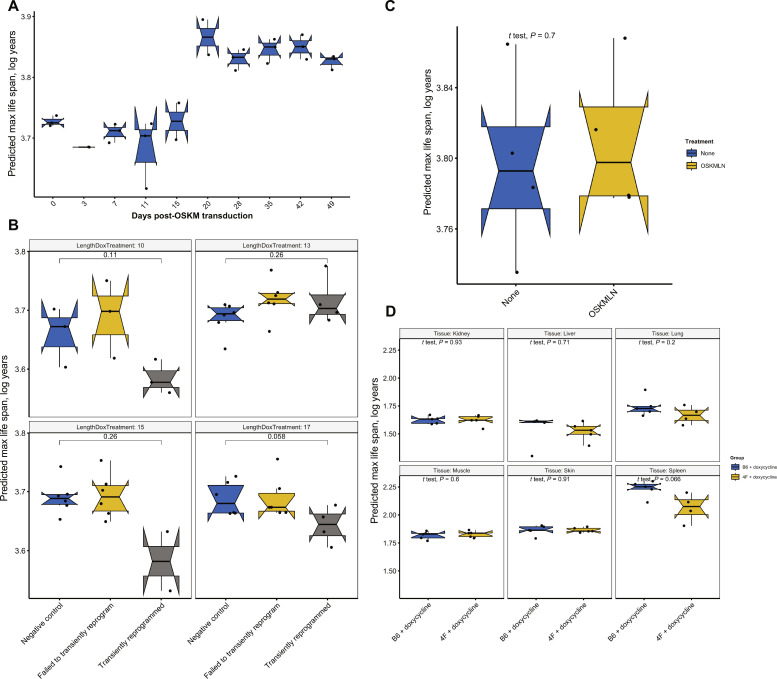
Partial or full OSKM reprogramming versus epigenetic maximum life span. (**A**) Predicted maximum life span in a 49-day full reprogramming time course of human dermal fibroblasts (HDFs) resulting in iPSCs (Kruskal Wallis test, *P* = 0.0086) (*56*). *Y* axis: log(maximum life span) calculated from DNAm arrays from the following cell populations: day 0 (HDFs), day 3 [OSKM expressing enhanced green fluorescent protein (EGFP) (+) HDFs], days 7 to 28 [TRA-1–60 (+) cells at intermediate stages of reprogramming], and iPSCs after day 35. (**B**) Predicted maximum life span of HDFs after transient reprogramming (GSE165179) (*37*). Different lengths of transient reprogramming were separated into subpanels. Negative control cells, transiently reprogrammed cells (CD13^−^ SSEA4^+^), and cells that failed to transiently reprogram (CD13^+^ SSEA4^−^) were included in the plot. (**C**) Predicted maximum life span of HDFs with transient expression of *Oct4*, *Sox2*, *KLF4*, *Myc*, *LIN28*, and *NANOG* (OSKMLN) (GSE142439) (*33*). OSKMLN was daily transfected for four consecutive days, and DNAm was measured 2 days after the interruption. (**D**) Predicted maximum life span in various tissues of 4F mice after 7 months of treatment (GSE190665) (*35*). B6 or 4F mice were given doxycycline in drinking water for 2 days followed by 5 days of withdrawal. The treatment started at 15 months of age and continued until 22 months of age (7-month treatment). B6 mice: WT mice; 4F mice: mice with the OSKM polycistronic cassette.

Our findings (Fig. 4) show the maximum life-span predictor outcomes for various cellular reprogramming treatment groups. Notably, human dermal fibroblasts subjected to a full reprogramming course based on OSKM transduction exhibited a slightly increased (and statistically significant *P* < 0.05) epigenetic maximum life span after 20 days (see Fig. 4A). Meanwhile, in a partial reprogramming experiment (GSE165179) (*37*), the treatment group displayed a marginally reduced mean predicted maximum life span. However, the disparity between the groups did not reach a statistically significant level (Fig. 4B).

We note that our examination of tissue and cell types did not yield conclusive evidence indicating a substantial divergence in the epigenetic maximum life span between embryonic stem cells or iPSCs and primary cells (see fig. S5). Our findings are somewhat inconclusive. Although full reprogramming in human dermal fibroblasts hints at an increase in epigenetic maximum life span after 20 days of OSKM administration, transient reprogramming experiments in humans and mice were unable to confirm this effect (Fig. 4, B to D). We discuss caveats surrounding the measurement platform below.

### Human epidemiological cohort studies

**Fig. 5.**
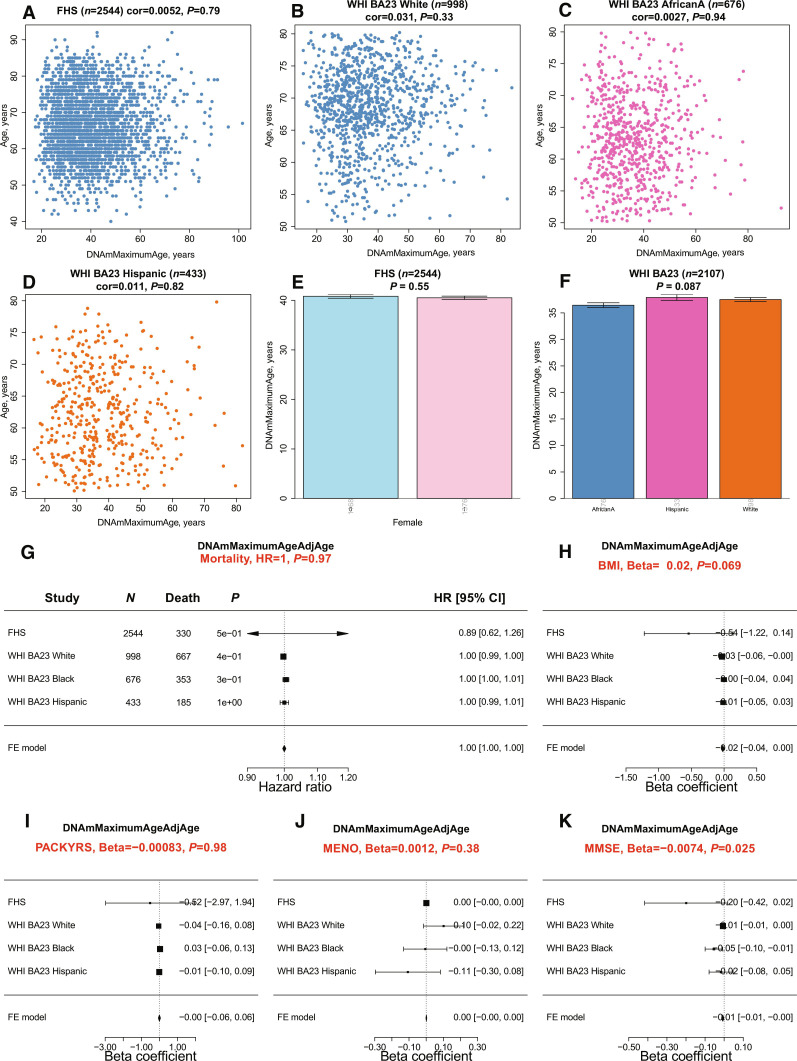
Methylation-based estimate of maximum life span in human cohorts. (**A** to **D**) Scatterplots of the predicted maximum life span, transformed from log-years back to years (DNAmMaximumLifespan, *x* axis), against chronological age (*y* axis). These panels depict data from (A) *n* = 2544 Caucasians of European ancestry in the FHS Offspring Cohort and (B to D) *n* = 2107 women from the Women’s Health Initiative Cohort Broad Agency Award 23 (WHI BA23). The data are further categorized by three racial/ethnic groups: European ancestry, African American ancestry (AfricanA), and Hispanic ancestry. Each data point symbolizes an individual and is color-differentiated based on ethnic group. Titles indicate the sample size and furnish the Pearson correlation coefficients accompanied by their respective *P* values. (**E**) contrasts DNAm maximum life span with sex in the FHS, while (**F**) relates to ancestry. (**G**) Forest plot summarizing a meta-analysis of Cox regression models for time to death (due to all causes), based on the age-adjusted version of DNAmMaximumAge. This analysis spans various study-ethnic groups, with each row detailing the hazard ratio (95% CI) for a 1-year elevation in DNAmMaxLifespanAdjAge. The title reports the meta *P* value, derived using inverse variance–weighted fixed-effect (FE) models. Forest plots showcase the correlation between age-adjusted DNAmMaxLifespan and the following variables: (**H**) human body mass index (BMI), (**I**) self-reported smoking pack-years (PACKYRS), (**J**), age at female menopause (MENO), and (**K**) Mini-Mental State Examination (MMSE) scores. The analysis, which spans different study-ethnic groups, outlines in each row the correlation coefficient (95% CI) corresponding to a 1-year increase in DNAmMaxAgeAdjAge. All *P* values are two-sided and are presented in their nominal form, without adjustment for multiple comparisons.

We used methylation-based estimators to assess the maximum life span in blood samples sourced from participants of the Framingham Heart Study (FHS) (*n* = 2544) (*38*) and the Women’s Health Initiative (WHI) (*n* = 2107) (*39*, *40*). Given that these samples were processed using a different methylation platform (the human Infinium 450K array), we used the Array Converter software to convert values from the mammalian methylation probes (*18*). We observed no significant correlations between the predicted maximum life span and the actual age of participants across three distinct racial/ethnic groups (Fig. 5, A to D). It is important to highlight that this finding contrasts with our previous analysis, where we identified a correlation between age and epigenetic maximum life span in human blood (*R *= 0.51, fig. S2N). These discrepancies likely arise from variations in measurement platforms. Our earlier analyses used the mammalian methylation array, whereas the epidemiological cohort studies used the human Illumina 450K array.

Our analysis reveals no significant associations with other demographic variables in blood samples, the DNAm-based maximum life span does not show a significant association with sex (*P* = 0.55, Fig. 5E), racial/ethnic group (*P* = 0.087, Fig. 5F), human mortality risk (*P* = 0.97, Fig. 5G), body mass index (*P* = 0.069), smoking pack years (Fig. 5I), or age at female menopause (*P* = 0.38, Fig. 5J). The Mini-Mental State Examination (MMSE) is a diagnostic tool for cognitive impairment and dementia. The MMSE evaluates various cognitive domains. A higher score on the MMSE indicates better cognitive functioning. We noted a marginally significant negative correlation (*P* = 0.025, Fig. 5K) between MMSE and age-adjusted DNAm life span. However, this significance disappears after accounting for multiple comparisons.

We delved into the relationship between our methylation-based life-span estimators and several dietary and health-related biomarkers (fig. S16). This comprehensive assessment covered 59 variables: 27 from self-reported dietary inputs, 9 from blood-based dietary measurements (including mean carotenoid levels, indicative of vegetable and fruit consumption), and 17 clinical indicators (including metabolic characteristics, central adiposity, inflammatory markers, leukocyte telomere length, cognitive performance, and lung function). We also analyzed lifestyle and demographic variables (diet, exercise, education, and income). Upon analysis, neither the epigenetic estimate of maximum life span nor its age-adjusted counterparts showed any significant association with the biomarkers after adjusting the analysis for multiple comparisons (fig. S16). The results suggest that lifestyle behaviors do not profoundly influence the maximum bounds of human life span, as measured by epigenetic predictors. However, it is essential to highlight a notable limitation in our analysis: the human data were sourced from a different methylation array platform and was heavily dependent on imputation methods. Future research should revisit these findings using data from methylation platforms that assess the highly conserved CpGs on the mammalian array.

### Evaluation in different dog breeds

**Fig. 6.**
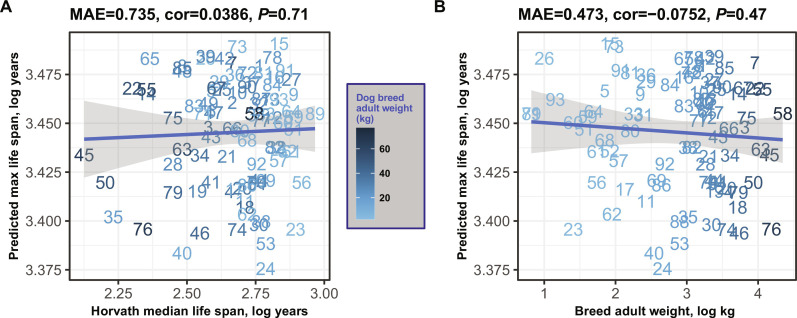
Mammalian life-span prediction applied to blood methylation data from 90 different dog breeds. All quantities are log-transformed (base e). Predicted log-transformed maximum life span (*y* axis) versus characteristics of dog breeds. (**A**) actual maximum life span of the breed (*x* axis). Maximum age of the dog breed was estimated as the product of 1.33 times the median life span of the breed from Horvath *et al*. (*41*). (**B**) Average adult weight of the dog breed. Each integer label corresponds to a different dog breed (*41*).

Dog breeds display a remarkable variability in life span, with certain breeds outliving others by as much as twofold. We assessed our epigenetic predictor of maximum life span using 742 individual blood samples sourced from 93 distinct dog breeds (*41*). However, our canine dataset presented two primary challenges. First, the representation of dogs within each breed was inconsistent, ranging from as few as 2 samples for the English Setter to as many as 95 for the Portuguese Water Dog. Second, there was a disparity in age distributions across breeds; for example, the relative ages *R* (defined as ratio of age divided by maximum lifespan of the species dog) for the Otterhound breed spanned from 0.06 to 0.14, while for the Beagle, *R* ranged from 0.06 to 0.73 (*41*). To average out these inconsistencies, we took the average of maximum life-span predictions for each breed. When applying the mammalian maximum life-span predictor to blood samples from 90 diverse dog breeds (Fig. 6), we did not observe a significant correlation between the predicted mammalian maximum life span and either the breed’s average/maximum life span or its average weight. Overall, these results suggest that the epigenetic predictor of mammalian life span is not effective in predicting breed-specific life spans in dogs.

The minimal variation in predicted maximum life span for different dog breeds (Fig. 6) may reflect a close alignment with the epigenetic life span of their wolf ancestors. This pattern indicates that the epigenetic predictor of maximum life span is not substantially influenced by the recent selective breeding by humans, but rather mirrors the life span of the dogs’ progenitor species. To examine this further, we developed another multivariate predictor of maximum life span that encompasses all mammalian species, including 93 dog breeds. This model was devised in a manner akin to that shown in Fig. 1 but with the following distinction: it replaces a single entry for *Canis lupus familiaris* with 93 individual breed-specific entries, thereby capturing the variance in life span across breeds (fig. S17A). The negligible Pearson correlation among dog breeds (*r* = 0.068, fig. S17B) highlights the inability of this predictor to effectively differentiate between breeds with longer and shorter life spans. Subsequently, we constructed a distinct multivariate predictor of breed life span trained solely on data from dog breeds. This specialized predictor shows a moderate correlation (*r* = 0.42, fig. S17C) for the log-transformed median life span of dog breeds. However, the modest nature of this correlation infers that methylation data may offer limited insight into the nuances of life span disparities among dog breeds.

## DISCUSSION

Drawing from the comprehensive dataset of our Mammalian Methylation Consortium, we developed multivariate predictors that adeptly discern maximum life span and associated life-history traits. Notably, our epigenetic estimator demonstrated heightened precision for gestation duration (*R* = 0.96) compared to maximum life span (*R* = 0.89). This discrepancy might be attributed to the inherent challenges in procuring accurate maximum life-span data across a diverse array of species.

In terms of sexual dimorphism in life-span predictions, for most species, there was a congruence in the predicted maximum life span between sexes. However, a distinct trend emerged in 17 species, including humans, where females displayed a longer predicted life span, with harbor seals being a notable exception. This observation resonates with previously published studies that underscore the longevity advantage of females (*42*–*44*).

Our epigenetic markers’ predictive capacity seems to add information beyond phylogenetic correlations, indicating their broader applicability. Neither chronological age nor typical adult weight appeared to markedly sway the accuracy of our life-history trait predictors. In numerous species, there was a conspicuous absence of correlation between chronological age and the epigenetic life span.

The actual maximum life span of humans, at 122.5 years, exceeds our epigenetic maximum life-span estimates. For humans, the highest epigenetic life-span values were observed in blood and epidermis samples, at 98.1 and 94.6 years, respectively. This trend of elevated epigenetic life span in blood samples is consistent across various species, from humans to brown rats. We did not find definitive evidence suggesting that the epigenetic maximum life span of embryonic stem cells or iPSCs notably diverges from that of somatic cells. The biological significance of cell type–and tissue-specific variations in epigenetic life-span predictions warrants further investigation. Epigenetic maximum life span showed little variation across dog breeds, indicating that it is not affected by recent genetic selection enforced on dogs and may represent the ancestral state of the dog as a species.

Our study on predicting maximum life span did not incorporate DNA sequence data. Instead, we concentrated on methylation levels at highly conserved CpG sites. Nonetheless, DNA sequence information holds promise for developing life-span predictors. For instance, Mayne *et al*. (*10*) introduced an effective predictor of maximum life span in vertebrates, using the density of CpG sites across 42 selected promoters, highlighting the use of DNA sequence data in multivariate prediction models (*10*). Although our focus on methylation contrasts with Mayne *et al*.’s (*10*) sequence-based approach, we similarly identified a connection to CpG islands, as detailed in our companion paper (*18*). The latter paper presents findings from epigenome-wide association studies (EWAS) on maximum life span, where individual cytosines’ correlation with log-transformed life span was examined. While a comprehensive discussion of the EWAS findings exceeds this paper’s scope, we highlight essential insights: CpG islands and related chromatin states, such as transcriptional start sites (TSS1) and flanking promoter states (PromF4, PromF5), show enrichment for CpGs negatively associated with maximum life span (*18*, *45*). In essence, species with longer life spans exhibit lower methylation levels at CpG islands compared to shorter-lived species.

Analysis of murine life-span interventions showed that only growth hormone knockouts showed extended epigenetic life span in liver and kidney tissues, while other tissues and long-lived strains did not influence epigenetic maximum life span. Similarly, caloric restriction did not affect epigenetic maximum life span.

Our analysis of human cohorts, despite its comprehensiveness, did not definitively determine the effects of lifestyle on epigenetic maximum life span. One possible constraint might arise from using different methylation array platforms for data gathering (specifically, the human Illumina 450K array as opposed to the mammalian methylation array). For more accurate insights in future human epidemiological cohort studies, it would be beneficial to profile the highly conserved CpGs using the mammalian methylation array.

Together our results suggest that species maximum life span is strongly associated with an epigenetic signature that is largely independent of sex, body mass, calorie restriction, or other lifestyle factors. This signature may be an intrinsic property of each species that is difficult to change. Only growth hormone knockout and full reprogramming had some effect on epigenetic maximum life span. It would be interesting to identify novel interventions that affect epigenetic maximum life span as they may be the key to achieving large life-span differences observed between species.

## MATERIALS AND METHODS

### Human subjects

We used existing data from the Mammalian Methylation Consortium (*18*). Detailed ethics statements can be found in the original citation (*18*). The secondary use of the other de-identified/coded human tissue samples (blood, postmortem tissues) is not interpreted as human subjects’ research under US Department of Health & Human Services 45 CFR 46. Therefore, the need to obtain written informed consent from human study participants was waived (secondary use of deidentified tissues). Human samples were covered by University of California Los Angeles IRB#18-000315.

### Animal research

All mice were maintained and bred under standard conditions consistent with National Institutes of Health guidelines and approved by the University of California, Los Angeles Institutional Animal Care and Use Committees. Additional and detailed ethics statements can be found in (*18*).

### DNAm data

We used existing data from the Mammalian Methylation Consortium that were published previously (*18*). All data were generated using the mammalian methylation array (HorvathMammalMethylChip40) (*16*), which provides high sequencing depth of highly conserved CpGs in mammals. Nearly 36,000 probes (cytosines) on the array exhibit high levels of sequence conservation within mammalian species (*16*). The subset of species for which each probe is expected to work is provided in the chip manifest file, which can be found at the National Center for Biotechnology Information (NCBI) Gene Expression Omnibus (GEO) as platform GPL28271, and on the Github webpage from the Mammalian Methylation Consortium. The SeSaMe normalization method was used to define β values for each probe and to calculate detection *P* values (*46*).

### Data description

We analyzed methylation data from 348 mammalian species representing 25 of 26 taxonomic orders (table S2 and Fig. 1). The only order not represented was the marsupial order Peramelemorphia. DNA was derived from 59 different tissues and organs including blood, skin, liver, muscle, and brain regions (table S1).

### Life-history traits and anAge database

The high accuracy of the epigenetic estimator of maximum life span is a testament to the success of a decade-long effort of biologists and the anAge database (*2*) to establish this elusive phenotype. For several species, maximum life span was not available in anAge. For select species, we used a *K* = 1 nearest neighbor predictor to impute values. Therefore, we limited our comparative analysis to species for which this value was available and did not require imputation. To enhance the reproducibility of our findings, we include our updated version of the anAge database (*2*) (table S1).

### Multivariate estimators of maximum life span

The regression coefficients from the final predictor, that is, the full model trained on all available species-level data for extrapolation purposes, are reported in table S5. For most species, relatively few animals informed the determination of maximum life span, which may bias this life-history trait (*47*, *48*).

To compensate for the disparity in data, with the maximum life span of humans and mice being derived from extensive studies and that of other mammals from fewer observations, we have applied a multiplicative correction factor of 1.3 to the maximum life-span values of the latter. This correction is based on the presumption that maximum life-span estimates from the AnAge database are, on average, 30% lower than the true potential for all species except humans and mice. This 30% increment is admittedly arbitrary, chosen for its consistency with the methodology of our previous work on a universal mammalian clock (*17*, *18*). Nonetheless, we acknowledge the necessity for future research to explore more empirically founded adjustment methods and to assess their impact on the predictive accuracy of our model.

Furthermore, in the final model trained on all species, we calibrated the predictor using the mean and SD. This calibration was done to align it with the biomarker’s distribution, matching the observed life-span data (*49*).

The distributions of mammalian life-history traits, both in general and within our dataset, show a pronounced skew toward higher values. This skewness is largely due to the exceptionally long life spans of certain species, such as humans and bowhead whales, compared to most mammals. To better meet the normality assumption of our regression models, we have applied a logarithmic transformation to all three life-history traits in this study.

We used elastic net regression to build different multivariate predictors of maximum life span, gestation time, and age at sexual maturity (*50*). To build a model on the basis of CpGs that are present/detectable in most species, we restricted the analysis to CpGs with significant median detection *P* values (false discovery rate < 0.05) (*51*) in 85% of the species. This resulted in a lower-dimensional dataset consisting of 17,032 CpGs.

We used three strategies for building maximum life-span predictors. The first strategy ignored tissue type. Here, all tissue samples from a given species were averaged, resulting in a single observation per species. To arrive at unbiased estimates of the predictive accuracy of life span and other predictors, we used a LOSO cross-validation analysis that iteratively trained the predictive model on all but one species. Next, the predictor was applied to the observations from the left-out species. By cycling through the species, we arrived at LOSO estimates for each species. The second strategy formed average values for each stratum defined by tissue type and species. For example, this analysis formed an average value for human blood (considered as one stratum). The second approach allowed us to study the influence of tissue type on life-span predictions. This second strategy shows similar prediction correlations in all three life-history traits (fig. S8).

Third, we also conducted a LOCO analysis as described in the following.

Conducting a comprehensive leave–one–taxonomic order–out cross-validation presented challenges. The primary issue was the unequal distribution of animals across taxonomic orders; for instance, Rodentia comprised 27% of all species, while many orders had fewer than 3% (table S2). To circumvent this, we adjusted the leave-one-order-out analysis. In larger taxonomic orders with over 20 species (like Rodentia, Artiodactyla, Chiroptera, Primates, Carnivora, and Eulipotyphla), we left out all species except two, representing the minimum and maximum life span. These two species functioned as a benchmark, tasking the predictor to estimate the life span for the entire taxonomic order based on limited data. Conversely, smaller taxonomic orders were left out completely as test sets. For instance, orders such as Dasyuromorphia, Microbiotheria, Sirenia, and Tubulidentata were represented only by a single species (table S2). This modified approach was termed the LOCO analysis. A predictor heavily influenced by neighboring species with close life spans, like the tree-based k-NN, would likely struggle with this methodology. Notably, as we used k-NN for imputing missing life-span observations for several species, life-span estimates naturally favor k-NN. Therefore, for this specific analysis, we relied on the original anAge database (*2*) that was devoid of imputed values.

It became clear that, while the k-NN life-span predictor showed a reasonable prediction correlation, it frequently provided static and deviant predictions for entire taxonomic orders (Fig. 2B). When faced with any test set, the algorithm often perceived the “nearest” species as the two specified in the LOCO training set, or occasionally species in a neighboring small order. This led to uniform estimates across a taxonomic order, making the algorithm less effective for diverse species or clades.

For assessing the sex difference in individuals’ DNAm maximum life-span prediction results, we chose to conduct two-sided Wilcoxon rank sum tests (*20*) instead of Student’s *t* tests, for the following considerations: (i) small sample sizes in some species’ tissue-sex strata; (ii) weak normality assumption in these small sample sizes; (iii) Wilcoxon rank sum test is a relatively more conservative test than Student’s *t* test (*52*); (iv) both Wilcoxon rank sum test and Student’s *t* test work in other strata in which normality can be assumed and larger sample sizes are present; and (v) to be consistent across all strata and species, Wilcoxon rank sum test was used for sex difference in DNAm life-span predictions.

### Random forest predictors of species and tissue

We specified 100 trees, with a bootstrap resampling limited to 200 per category, to counteract the imbalance in tissue and species category sample sizes. Other settings are standard (default parameter choices) for our random forest predictors of species, tissue, and sex (the predictive performance is detailed in Table 1).

To discern which CpGs hold the greatest predictive value for species identification in our random forest model (Table 1), we used the Gini index–based measure of variable importance. To demonstrate the distinctiveness of our random forest species predictor from our multivariate model predictor of maximum life span (which relies on 152 CpGs), we highlight an interesting contrast. Among the top 100 most significant CpGs identified by the random forest predictor of species, only 2 overlap with the 152 CpGs used in the maximum life-span predictor (fig. S18). Furthermore, only 3 of the 152 CpGs are ranked within the top 1000 by the random forest predictor, underscoring the uniqueness of the predictors.

### Interventions in mice

We used existing mammalian methylation data from mouse studies (*18*). The mammalian array data were generated using two versions of the mammalian array: the original mammalian array (called “40K” array) and the expanded array (referred to as “320K”) that also includes mouse probes (*16*). Some CpG probes unique to each array required imputation. Methylation levels of CpG sites missing on the 320K array were imputed from median β values of the training mouse samples (“40K” array). None of the samples from the murine anti-aging studies were incorporated into the training set. Our DNAmMaxAge was assessed using the following independent test datasets: (1) Snell dwarf mice (*n* = 95), (2) GHRKO experiment 1 (*n* = 71), (3) liver specific GHRKO experiment 2 (*n* = 96), and (4) calorie restriction (*n* = 95).

*t* tests evaluated whether these conditions affected epigenetic maximum life span. The DNAm data from datasets 1 and 3 were collected using the Mammalian Methylation 320k customized array (available in GSE223943 and GSE223944). Datasets 2 and 4 are available at GSE223748. Below is a brief overview of the experiments. Comprehensive details can be found in the supplementary materials of (*18*).

Snell dwarf experiment (*n* = 95): We analyzed tissues from 47 Snell dwarfs and 48 age-matched wild-type control mice, aged around 6 months. Snell dwarf mice, known for an approximately 30 to 40% extended life span, lack growth hormone, thyroid-stimulating hormone, and prolactin. Methylation profiling was conducted on blood, cerebral cortex, liver, kidney, spleen, and tail from these mice.

GHRKO experiments: We analyzed tissues from full-body (*n* = 71) and liver-specific (*n* = 96) GHRKO studies. The full-body GHRKO mice exhibited prolonged life span, while liver-specific GHRKO did not. DNAm profiles were created for various tissues, and age matching was performed.

Calorie restriction study (*n* = 95): This study involved analyzing liver samples from 95 male mice, 59 from the calorie-restricted group, and 36 controls. All mice, sourced from UT Southwestern Medical Center, Dallas, were 1.57 years old and from the C57BL/6J strain.

### Cancer risk in different mammals

We sourced estimates of mammalian cancer risk from a recent study (*21*). Two key metrics were considered: First, cancer mortality risk (CMR)—this refers to the ratio of cancer-related deaths to the total number of individuals for whom postmortem pathological records exist. It is a measure that has been used in various comparative studies (*53*, *54*). Second, cumulative incidence of cancer mortality (ICM)—this metric gauges the risk of cancer mortality by eliminating potential biases from both left and right censoring. Notably, there is a strong correlation between CMR and ICM, with a Pearson correlation coefficient of *r* = 0.89 (*21*). However, neither of these metrics showed any correlation with epigenetic maximum life span.

### Mortality analysis in human epidemiological cohort studies

We estimated DNAm maximum age in blood methylation data from 4651 individuals from (i) the FHS offspring cohort (*n* = 2544 Caucasians, 54% women) (*38*) and (ii) WHI cohort (*39*, *40*) (*n* = 2107, 100% women). Since these data were generated on a different platform (the Ilumina 450K array), we applied the Array Converter algorithm to impute mammalian methylation array data (*18*). Although the epigenetic maximum life-span estimates are not correlated with chronological age, we defined a measure of epigenetic age acceleration (AgeAccel) as the raw residual resulting from regressing DNAm maximum life span on chronological age. By definition, the resulting DNAmMaxLifespanAdjAge measure is not correlated with chronological age. We applied Cox regression analysis for time to death (as a dependent variable) to assess if individual variation in the predicted maximum life span is attributable to mortality risk. The analysis was adjusted for age at blood draw and sex in the FHS cohort. We stratified the WHI cohort by ethnic/racial groups and combined a total of four results across the FHS and WHI cohorts using fixed-effect models weighted by inverse variance. The meta-analysis was performed using the metafor function in R.

### Dog breeds

We used existing methylation profiles from 742 blood samples, representing 93 distinct dog breeds (*C. lupus familiaris*) (*41*). Breed weight and average life-span data were compiled from multiple sources as outlined in (*41*). We formed consensus values by integrating information from the American Kennel Club and the Atlas of Dog Breeds of the World. Life-span approximations were derived from averaging standard breed life spans across sexes. This information was gathered from a myriad of publications, most of which are multibreed studies focusing on age and mortality causes from veterinary clinics, as well as extensive breed-specific research typically conducted by purebred dog associations. The specific sources for each breed’s median life span are cited in (*41*).

To derive a reliable estimate for the maximum life span of each breed, we based our calculations on the breed’s median life span. Specifically, we used the following formula: MaxLifespan = 1.33 × MedianLifespan. Notably, our conclusions hold even when applying different multipliers beyond 1.33, as the log transformation converts these multipliers into additive shifts. Comprehensive data on the breeds can be found in (*41*). Among the 93 breeds studied, median life spans varied between 6.3 years (Great Dane, with an average adult weight of 64 kg) and 14.6 years (Toy Poodle, average adult weight being 2.3 kg).

## Supplementary Materials

### This PDF file includes:

Figs. S1 to S18 Legends for tables S1 to S11 References
