# Supplementary material for: Epigenetic predictors of species maximum life span and other life-history traits in mammals
Source: Sci Adv. 2024 Jun 7;10(23):eadm7273. doi: 10.1126/sciadv.adm7273 (PMC11160467; doi:10.1126/sciadv.adm7273)
Supplement: Supplementary file 1 — Figs. S1 to S18 Legends for tables S1 to S11 References [file sciadv.adm7273_sm.pdf]

Science Advances
AAAS

Supplementary Materials for

Epigenetic predictors of species maximum life span and other life-history traits in mammals

Caesar Z. Li *et al.*

Corresponding author: Steve Horvath, shorvath@mednet.ucla.edu

Sci. Adv. **10**, eadm7273 (2024)
DOI: 10.1126/sciadv.adm7273

The PDF file includes:

Figs. S1 to S18
Legends for tables S1 to S11
References

Other Supplementary Material for this manuscript includes the following:

Tables S1 to S11

Supplementary Materials

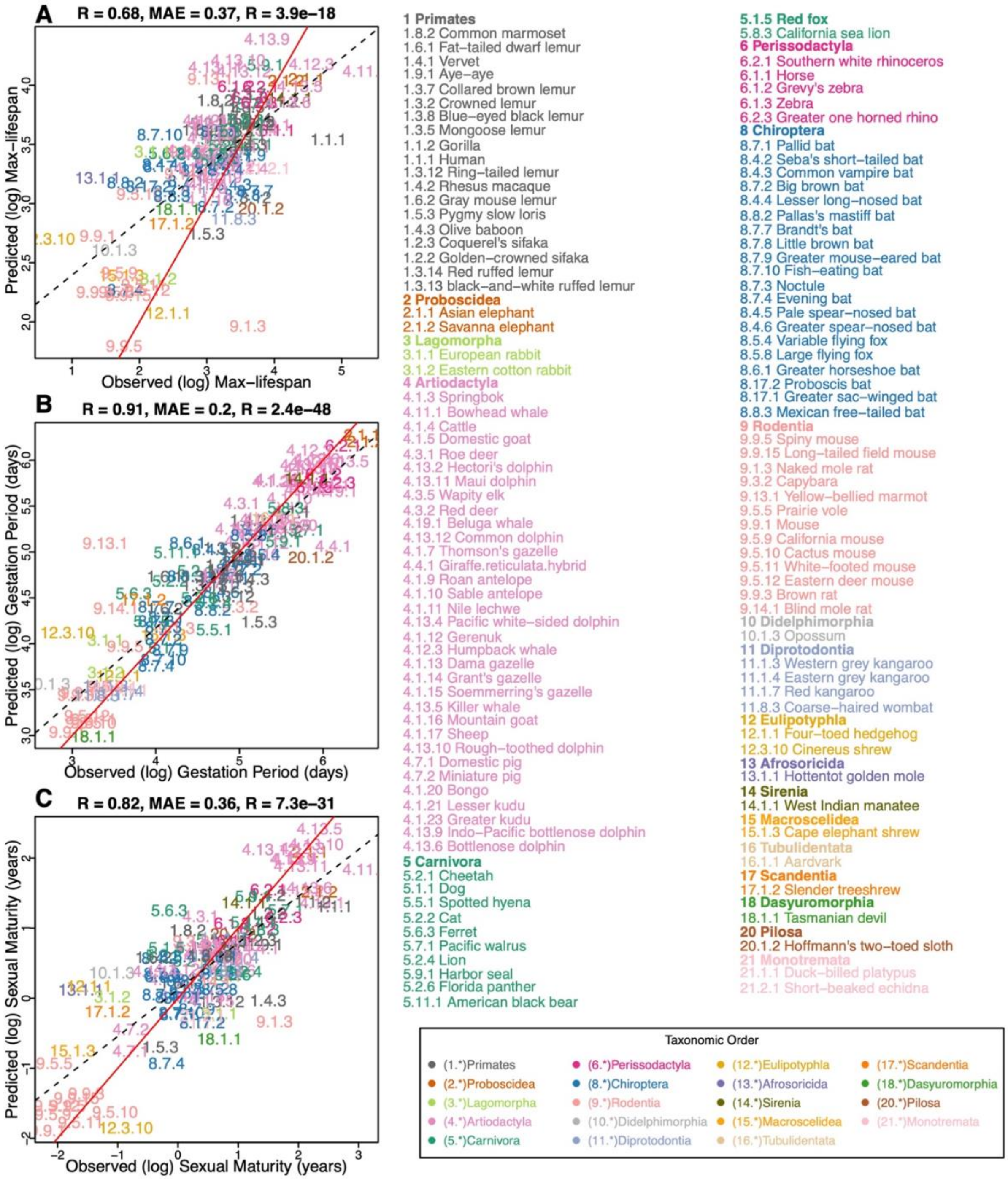

Fig. S1 | ElasticNet predictor based on young samples.

Elastic Net Predictor, Leave-one-species-out analysis, fitted on a subset of all young samples (species n = 122). Young samples are defined as samples whose age is both younger than five years and less than the species' average age at sexual maturation. Feature filtering and Elastic Net tuning parameter set-up is the same as those for **Fig. 1**. Three panels show predictors for **A,** log maximum lifespan (in log years), **B**, log-transformed gestation time (in log days), and **C**, log-

transformed age at sexual maturity (in log years). As with the **Fig. 1**, species appear as designated numbers in scatter plot panels; the corresponding common names and phylogenetic orders are annotated in Figure legends; as indicated by the taxonomic order legend, the whole number (number before the decimal separator) part of each mammalian number is assigned in accordance with the corresponding taxonomic order. Median absolute errors (MAE) from the regression errors; r and p denote Pearson's correlation and p-values, respectively. Numbers and colors are the mammalian species number and order annotation consistent with those of other Figures. Numeric values can be found in **table S1**. Red solid line represents the perfect prediction line, and the dotted line represents the fitted linear regression line.

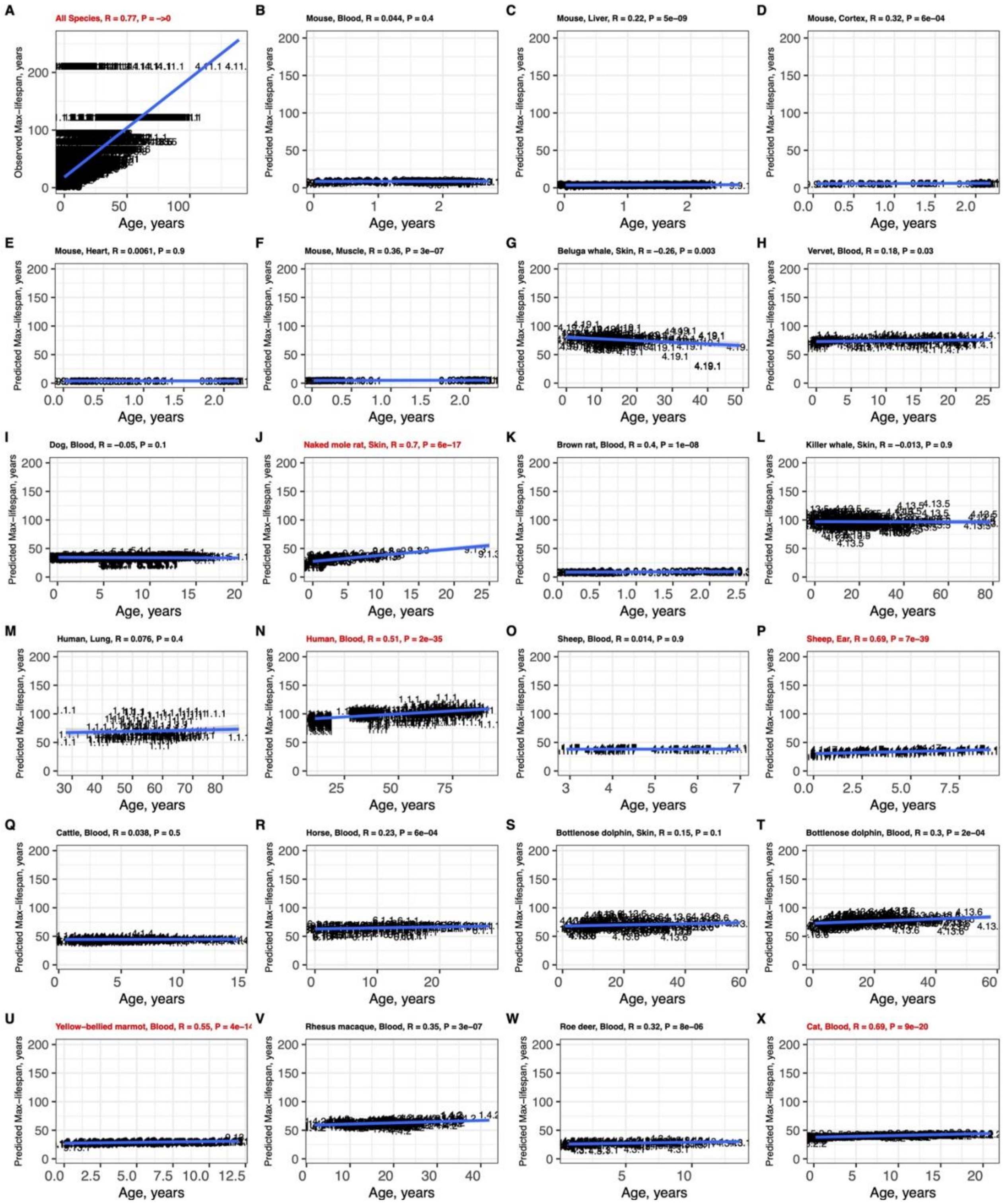

Fig. S2 | The maximum lifespan predictor applied to individual samples in comparison to their chronological ages.

Mammalian maximum lifespan predictor, based on averaged species methylation, was used to predict individual sample lifespans (in years scale). The predicted values are also stratified by species and tissues. Only species with >100 sample sizes are shown. To demonstrate natural relations between maximum lifespan and chronological age, panel **A** scatter plot shows

association between observed maximum lifespan and chronological age of corresponding samples. Each of panels **B–X** shows scatter plots of predicted lifespans converted to original scales vs. chronological age in specific species/tissue combinations. Numbers are the mammalian species number consistent with those in **fig. S1**. Red font is used when the absolute value of the Pearson correlation exceeds 0.5. Numeric values can be found in **table S1**.

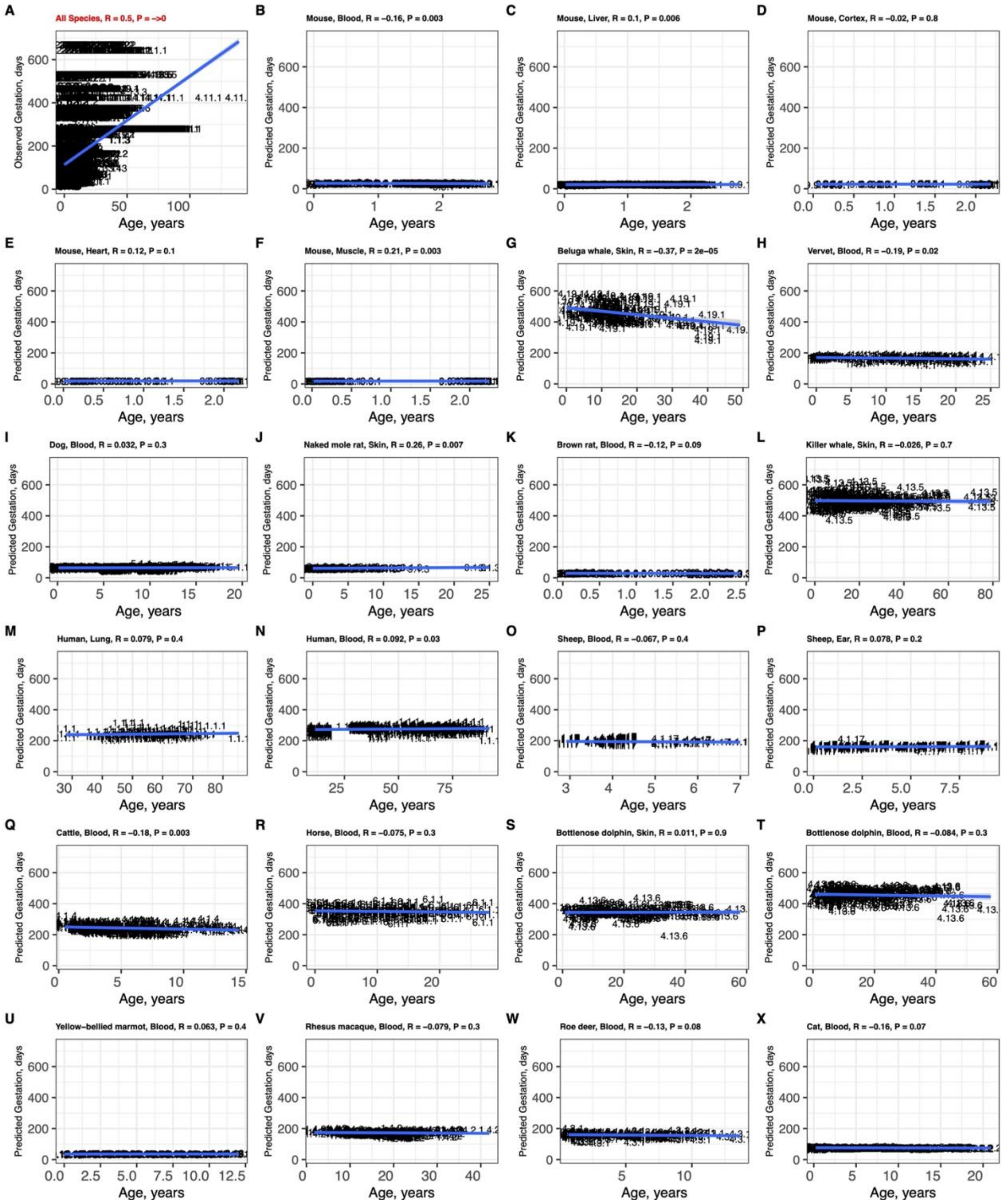

Fig. S3 | The gestation time predictor applied to individual samples in comparison to their chronological ages.

Gestation time predictor, based on averaged species methylation, was used to predict individual sample gestation time (in log days). The predicted values are also stratified by species and tissues. Only species with >100 sample sizes are shown. To demonstrate natural relations between gestation time (days) and chronological age, panel **A** scatter plot shows association between

observed gestation time (days) and chronological age of corresponding samples. Each of panels **B–X** shows scatter plots of predicted gestation time in log-days converted back to days vs. chronological age in specific species. Numbers are the mammalian species number consistent with those in **fig. S1**. Numeric values can be found in **table S1.3**.

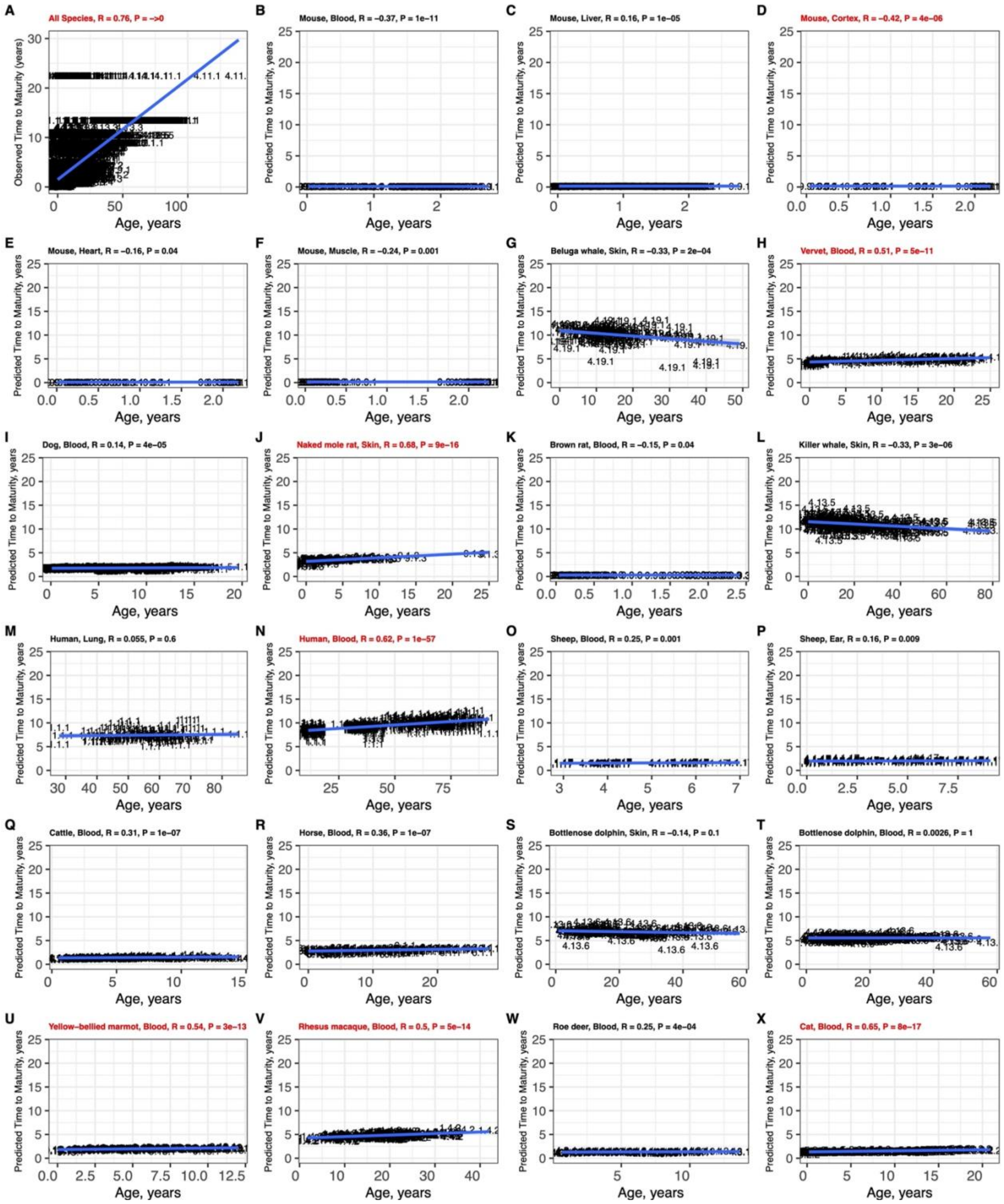

Fig. S4 | The time to sexual maturity predictor applied to individual samples in comparison to their chronological ages.

Time to sexual maturity predictor, based on averaged species methylation, was used to predict individual sample time to sexual maturity (in log years). The predicted values are also stratified by species and tissues. Only species with >100 sample sizes are shown. To demonstrate natural relations between time to sexual maturity and chronological age, panel **A** scatter plot shows

association between time to sexual maturity (years) and chronological age of corresponding samples. **B–X,** scatter plots of predicted age at sexual maturity in log-years converted back to years vs. chronological age in specific species. Numbers are the mammalian species number consistent with those in **fig. S1**. Numeric values can be found in **table S1.3**.

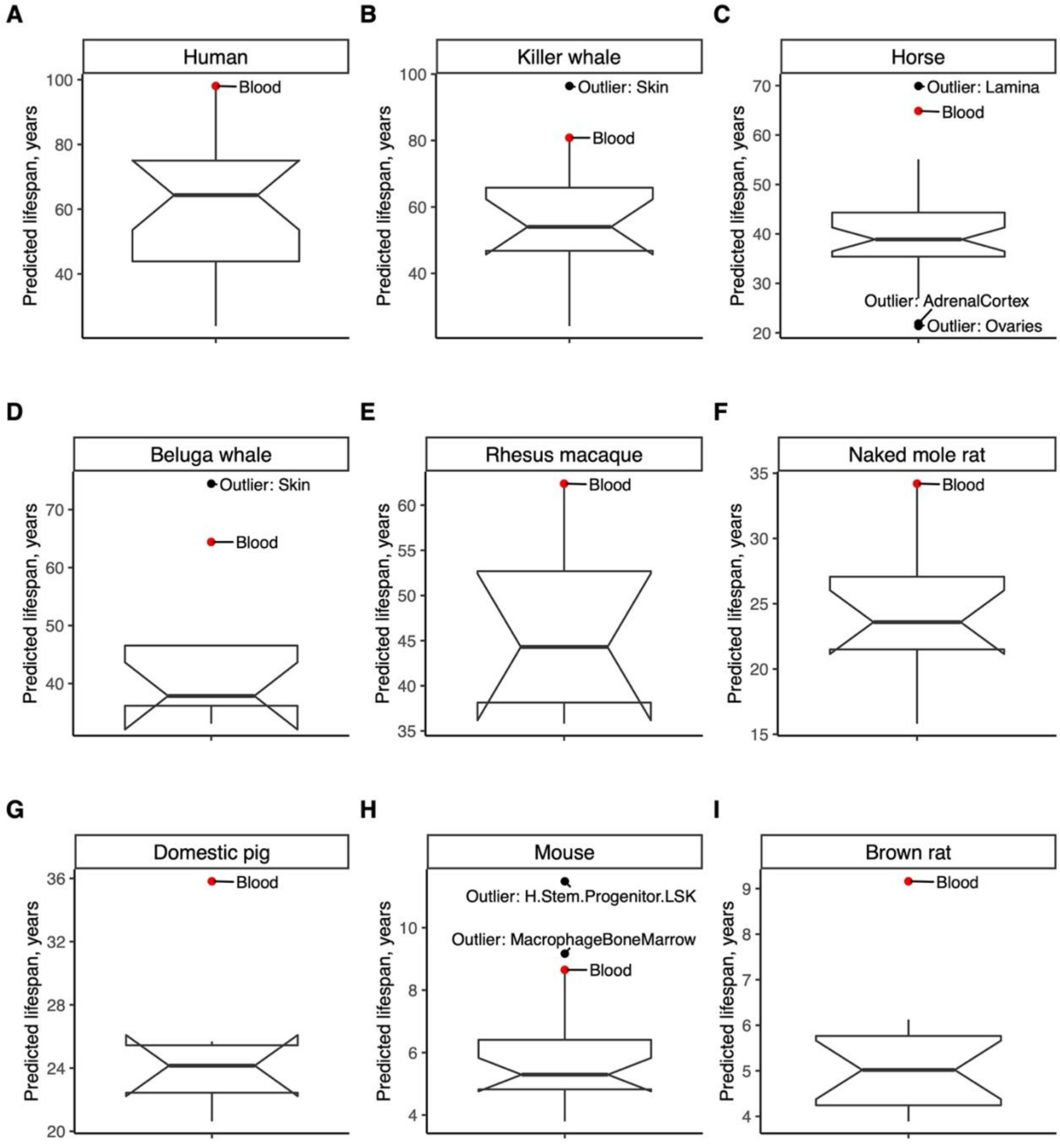

Fig. S5 | Tissue group differences in predicted mammalian maximum lifespan.

Tissue-agnostic predictor of mammalian maximum lifespan, based on averaged species methylation, was used to predict individual maximum lifespan (in log years). The predicted values are aggregated by taking the mean lifespan predictions by tissue groups. Panels **A-I** convert log scale back to original units (lifespan in years); only species with more than 6 different tissue types are shown; mean tissue predicted value outliers are annotated; Tissue type "H.Stem.Progenitor.LSK" stands for Lin (negative), stem cell antigen-1 (positive) receptor tyrosine kinase c-Kit (positive), which are cell surface markers to characterize a subset of cells within the bone marrow that is capable of self-renewal and differentiation into all types of

blood cells. **C**, Apart from blood, laminae are an outlying tissue in horses. Laminae are interlocking leaf-like tissues that connect the inner surface of the horse's hoof wall to the bone of the foot. The boxplot, as implemented in the R programming language, provides a visual summary of key statistics from a dataset: The median is represented by the horizontal line inside the box. The interquartile Range (IQR) encompasses the middle 50% of the data. The box's upper boundary represents the 75th percentile, while the lower boundary represents the 25th percentile. The IQR is the difference between these two values. The whiskers extend to the most extreme data point which is no more than 1.5 times the interquartile range from the box.

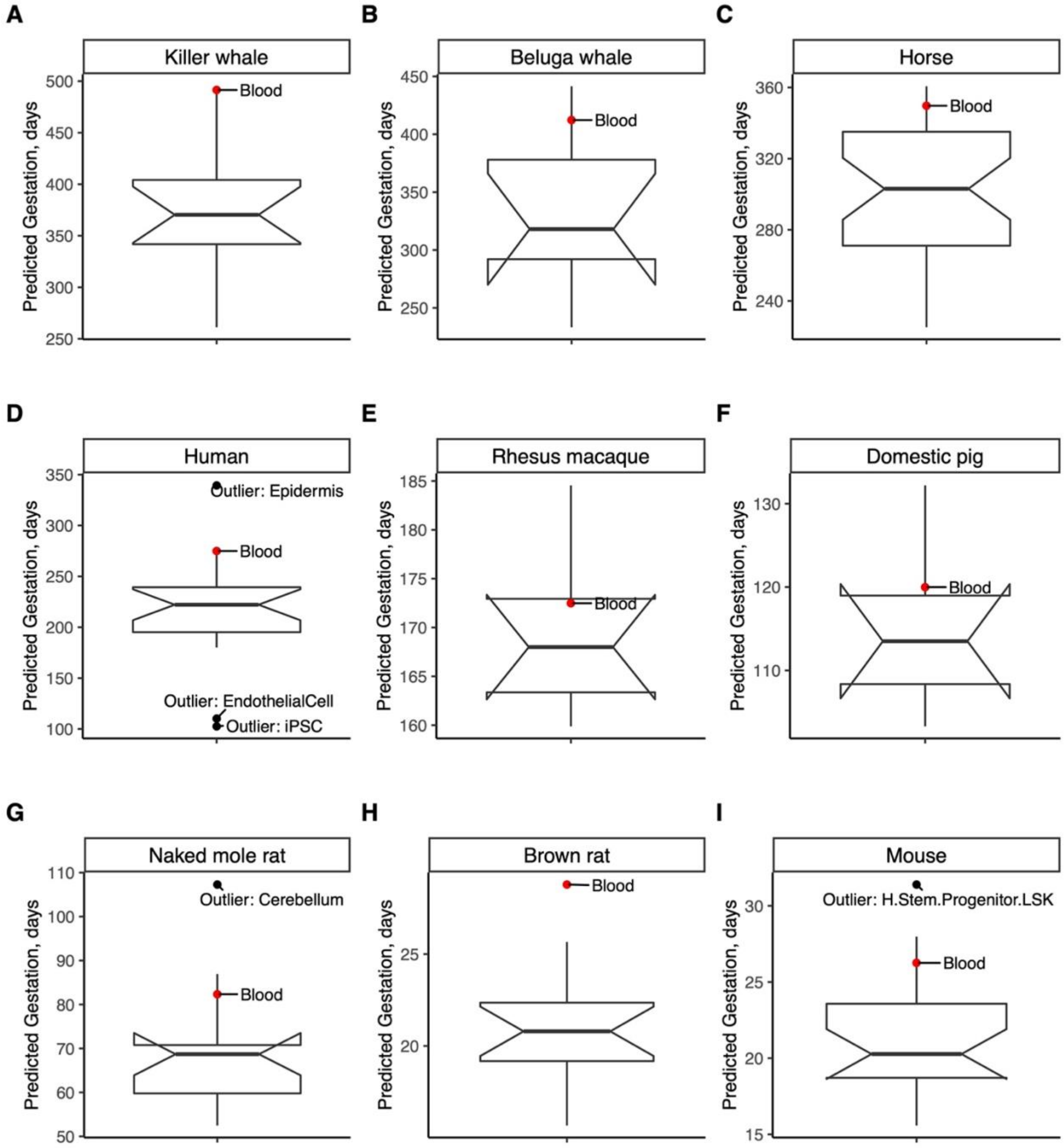

Fig. S6 | Tissue groups differences in predicted mammalian gestation time.

Tissue-agnostic predictor of gestation time, based on averaged species methylation, was used to predict individual sample gestation time (in log days). The predicted values are aggregated by taking the mean gestation time predictions by tissue groups. Panels **A-I** convert log scale back to original units (gestation in days); only species with more than 6 different tissue types are shown; mean tissue predicted value outliers are annotated; Tissue type “H.Stem.Progenitor.LSK” stands for “LSK Progenitor Hematopoietic Stem cells.” The boxplot, as implemented in the R programming language, provides a visual summary of key statistics from a dataset: The median is represented by the horizontal line inside the box. The interquartile Range (IQR) encompasses the middle 50% of the data. The box's upper boundary represents the 75th percentile, while the lower

boundary represents the 25th percentile. The IQR is the difference between these two values. The whiskers extend to the most extreme data point which is no more than 1.5 times the interquartile range from the box.

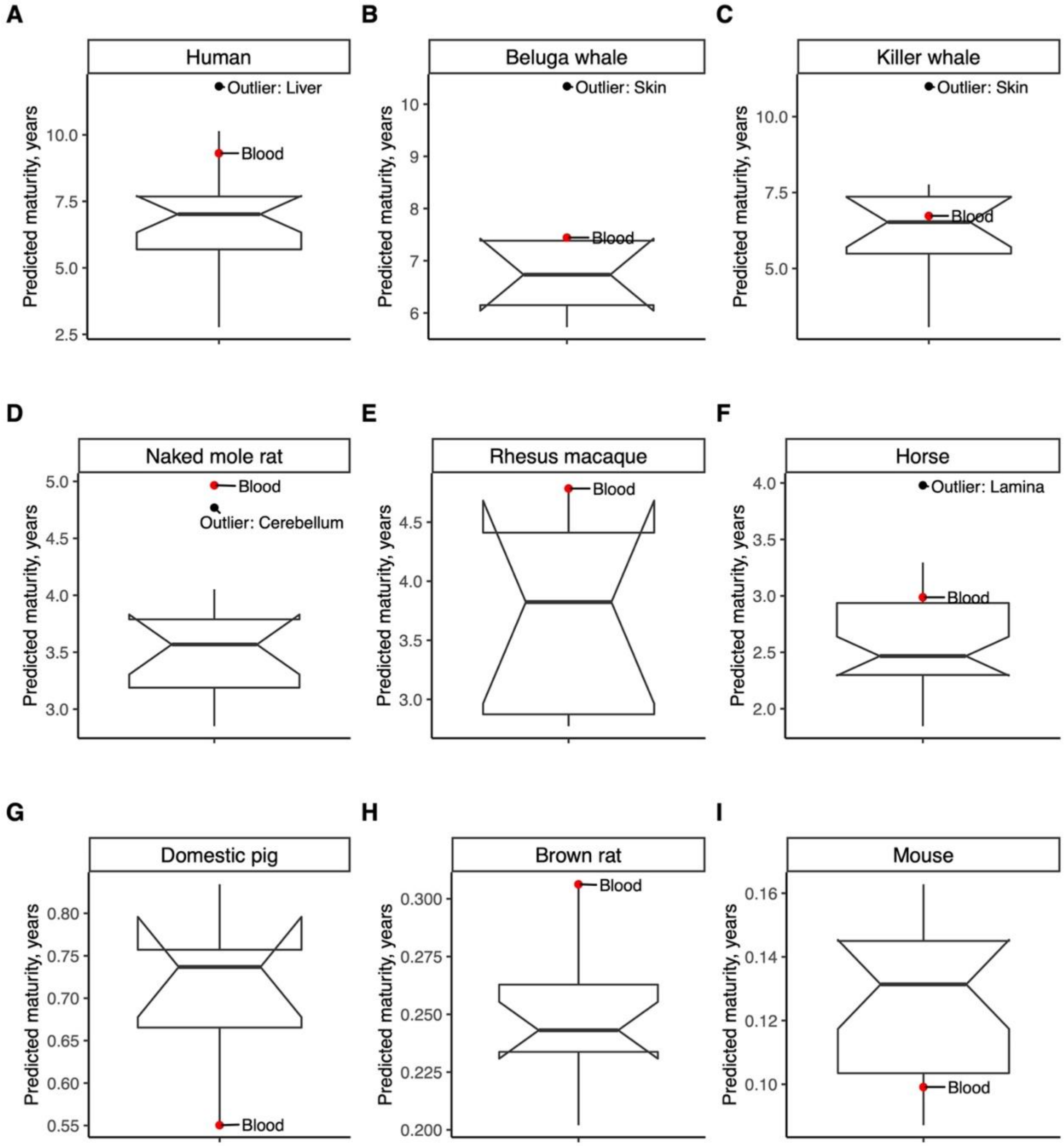

Fig. S7 | Tissue groups differences in predicted mammalian time to sexual maturity.

Tissue-agnostic predictor of time to sexual maturity. The boxplot shows median predicted values (short horizontal line) across tissue types. Significantly outlying tissues have been highlighted. The boxplot, as implemented in the R programming language, provides a visual summary of key statistics from a dataset: The median is represented by the horizontal line inside the box. The interquartile Range (IQR) encompasses the middle 50% of the data. The box's upper boundary represents the 75th percentile, while the lower boundary represents the 25th percentile. The IQR is the difference between these two values. The whiskers extend to the most extreme data point which is no more than 1.5 times the interquartile range from the box. Tissue-agnostic predictor of

time to sexual maturity predictor, based on averaged species methylation, was used to predict individual sample time to sexual maturity (in log years). The predicted values are aggregated by taking the mean lifespan predictions by tissue groups. Panels **A-I** convert log scale back to original units (age at sexual maturity in years); only species with more than 6 different tissue types are shown; mean tissue predicted value outliers are annotated; Tissue type "H.Stem.Progenitor.LSK" stands for "LSK Progenitor Hematopoietic Stem cells."

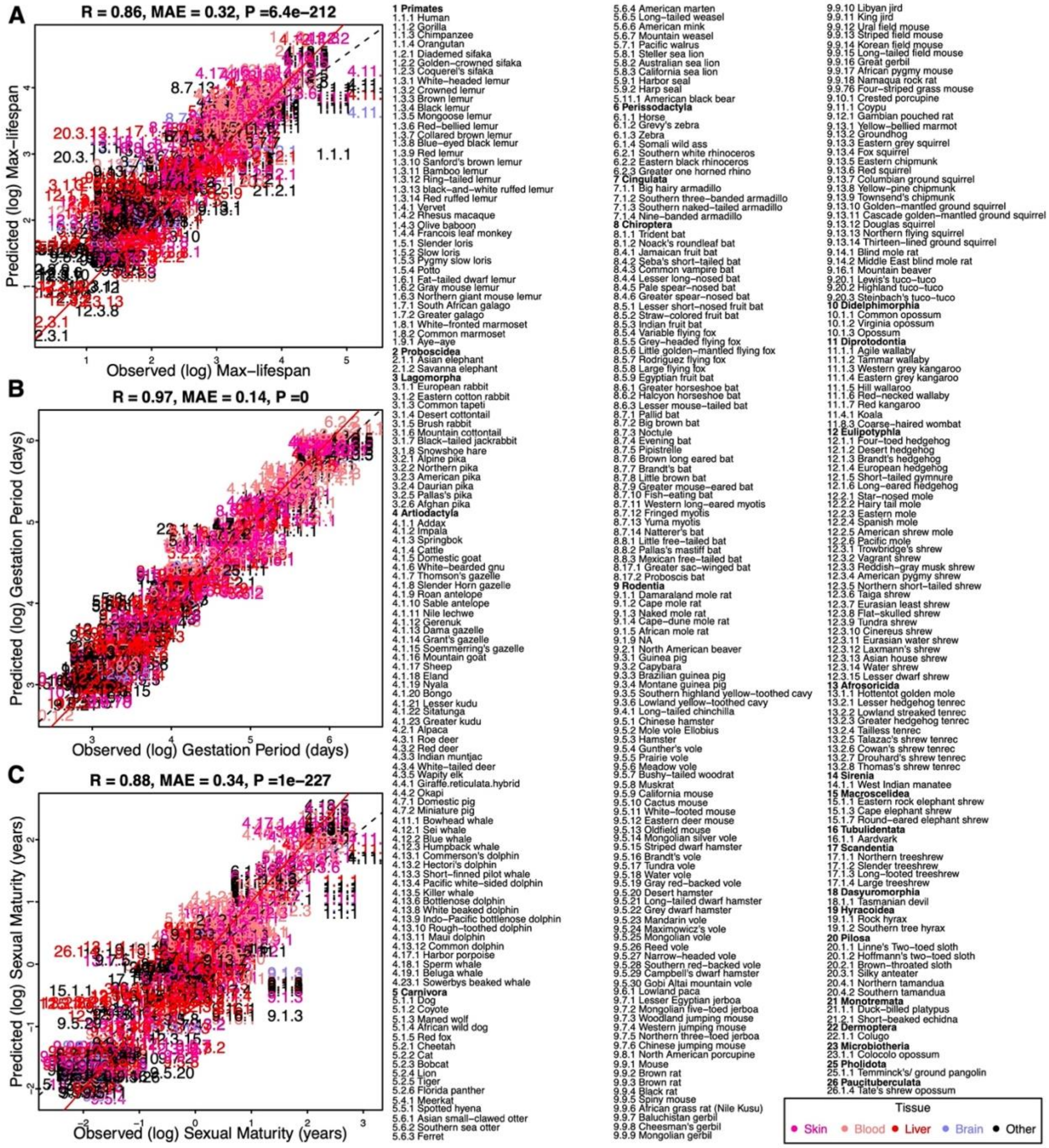

Fig. S8 | Tissue-aware predictors trained on species-tissue combinations.

A penalized joint linear model used to predict species lifespan (Elastic Net). Same framework as that of **Fig. 1**, except that it distinguishes tissue types. CpG probes are averaged by each species-tissue combination. Different tissues within the same species share the same maximum lifespan but retain different methylation levels. Three panels show predictors for **A,** log maximum lifespan (in log years), **B,** log-transformed gestation time (in log days), and **C,** log-transformed age at sexual maturity (in log years). Designated Mammalian numbers in scatter plot panels and the Figure legend are the same as those of main **Fig. 1.** MAE abbreviates median absolute errors from the regression errors; r and p denote Pearson's correlation and p-values, respectively. Numbers and colors are the mammalian species number and order annotation consistent with those of other Figures. Numeric values can be found in **table S3**. As with the **Fig. 1**, species appear as

designated numbers in scatter plot panels; the corresponding common names and taxonomic orders are annotated in Figure legends; the whole number (number before the decimal separator) part of each mammalian number is assigned in accordance with the corresponding taxonomic order. Red solid line represents the perfect prediction line, and the dotted line represents the fitted linear regression line.

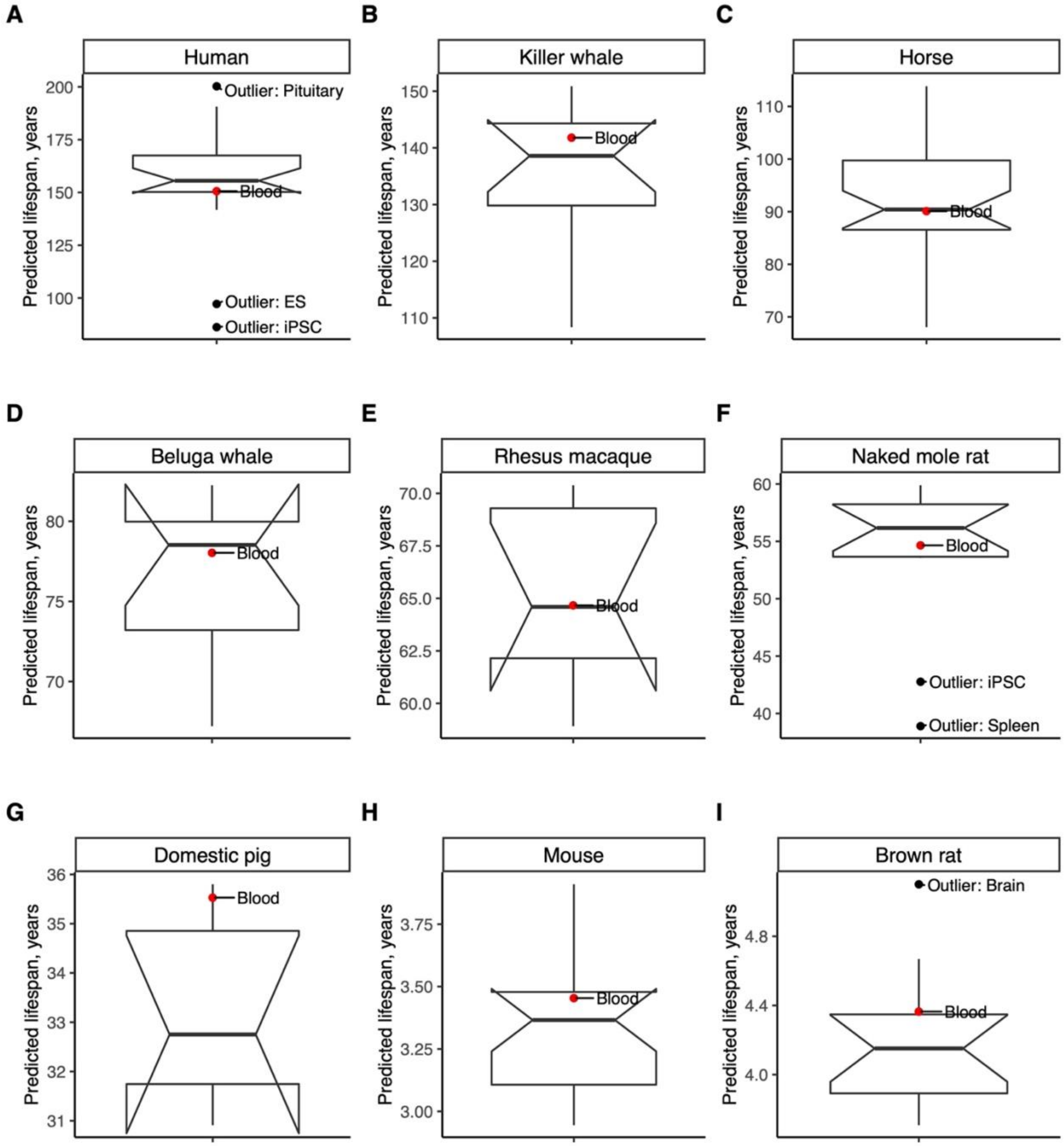

Fig. S9 | Tissue group differences in predicted mammalian maximum lifespan – Tissue-Aware

Tissue-aware predictor of mammalian lifespan, based on averaged species methylation, was used to predict individual sample lifespan (in log years). The predicted values are aggregated by taking the mean lifespan predictions by tissue groups. Panels **A-I** convert log scale back to original units (lifespan in years); only species with more than 6 different tissue types are shown; mean tissue predicted value outliers are annotated; Tissue type "H.Stem.Progenitor.LSK" stands for "LSK Progenitor Hematopoietic Stem cells."

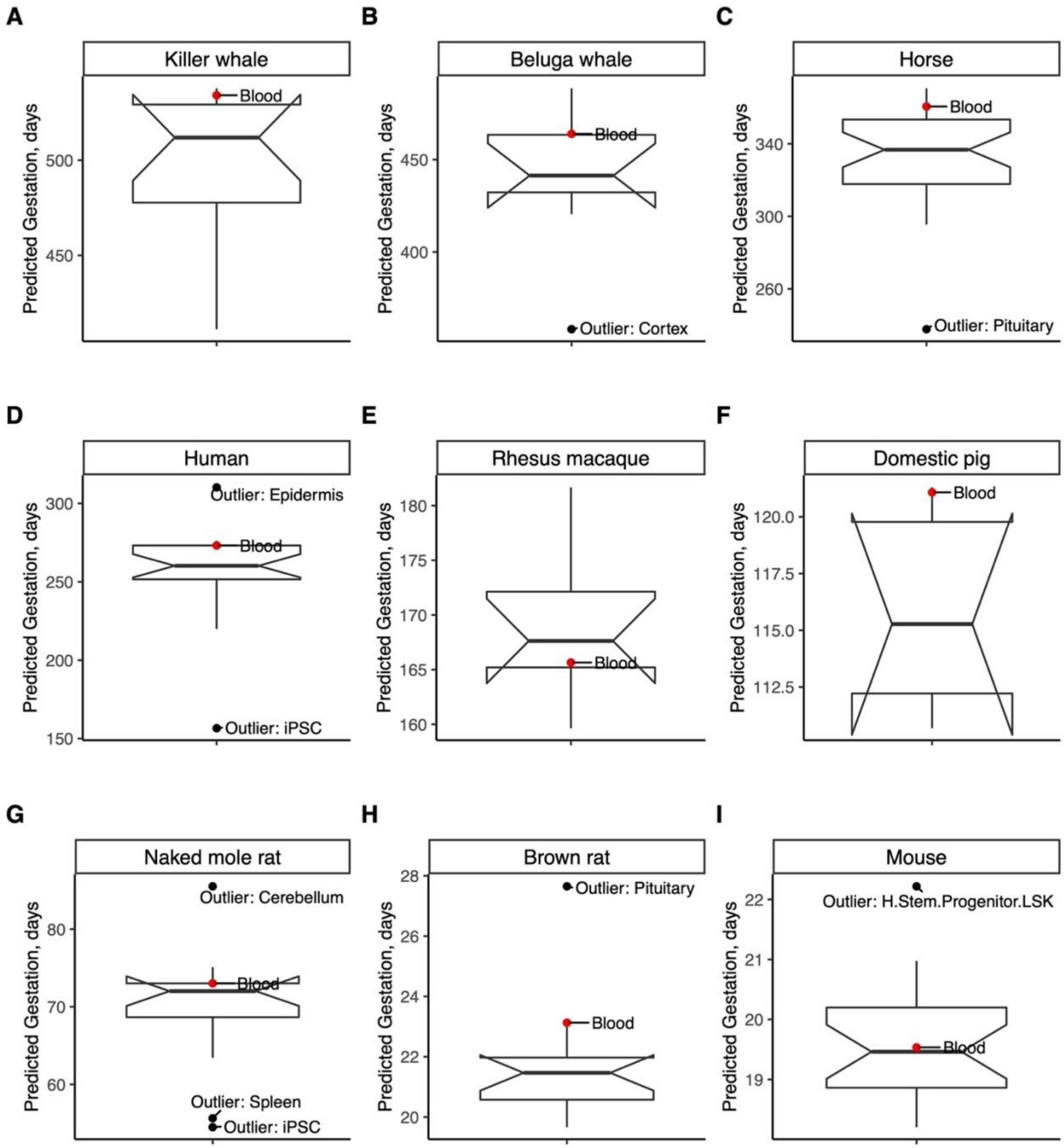

Fig. S10 | Tissue groups differences in predicted mammalian gestation time – Tissue-aware.

Tissue-aware predictor of gestation time, based on averaged species methylation, was used to predict individual sample gestation time (in log days), trained on tissue-aware data. The predicted values are aggregated by taking the mean gestation time predictions by tissue groups. Panels **A-I** convert log scale back to original units (gestation in days); only species with more than 6 different tissue types are shown; mean tissue predicted value outliers are annotated; Tissue type "H.Stem.Progenitor.LSK" stands for "LSK Progenitor Hematopoietic Stem cells."

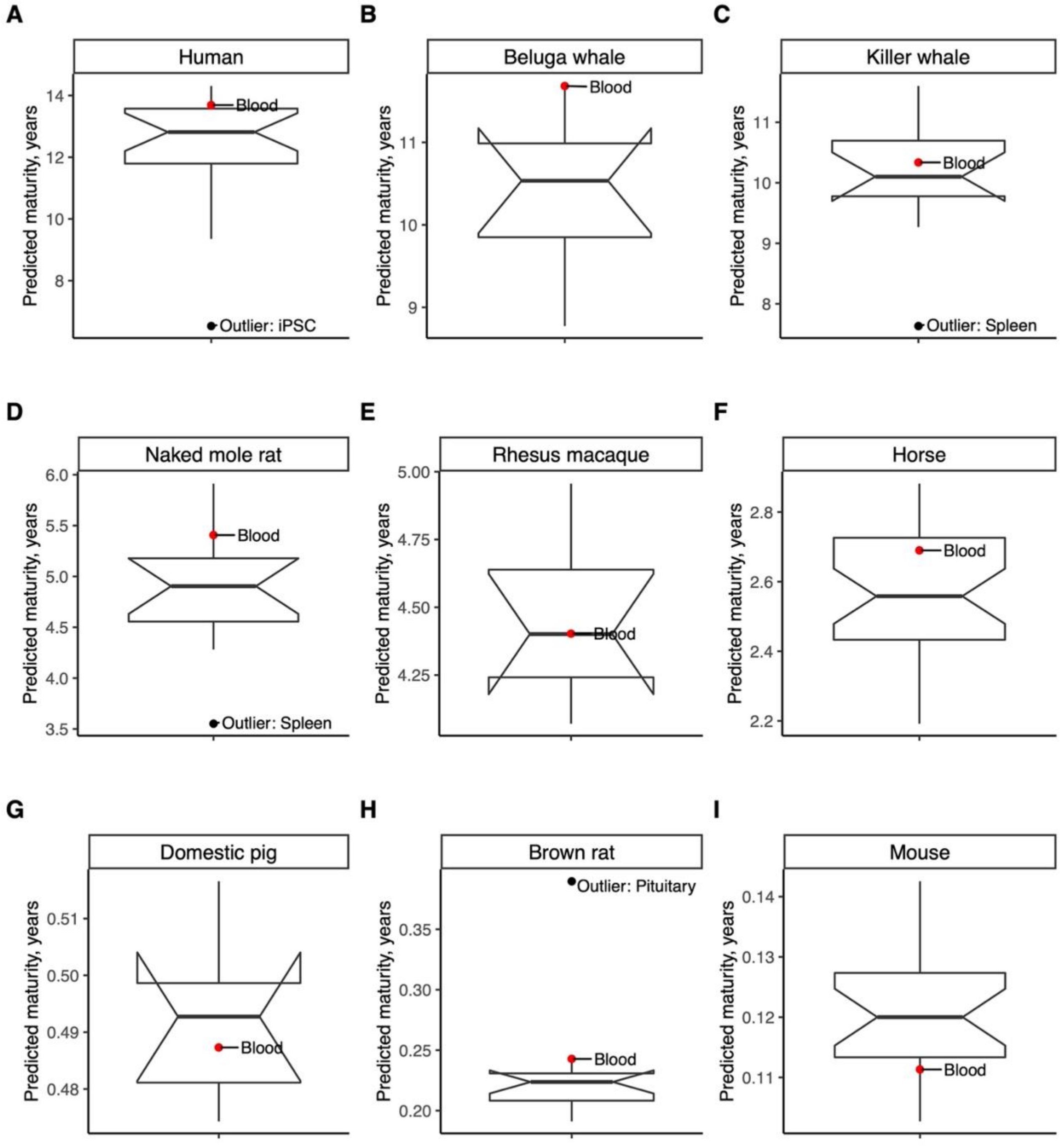

Fig. S11 | Tissue groups differences in predicted mammalian sexual maturity time – Tissue-aware.

Mammalian times to sexual maturity predictor, based on averaged species methylation, was used to predict individual sample time to sexual maturity (in log years), trained on tissue-aware data. The predicted values are aggregated by taking the mean lifespan predictions by tissue groups. Panels **A-I** convert log scale back to original units (age at sexual maturity in years); only species with more than 6 different tissue types are shown; mean tissue predicted value outliers are annotated; Tissue type “H.Stem.Progenitor.LSK” stands for “LSK Progenitor Hematopoietic Stem cells.

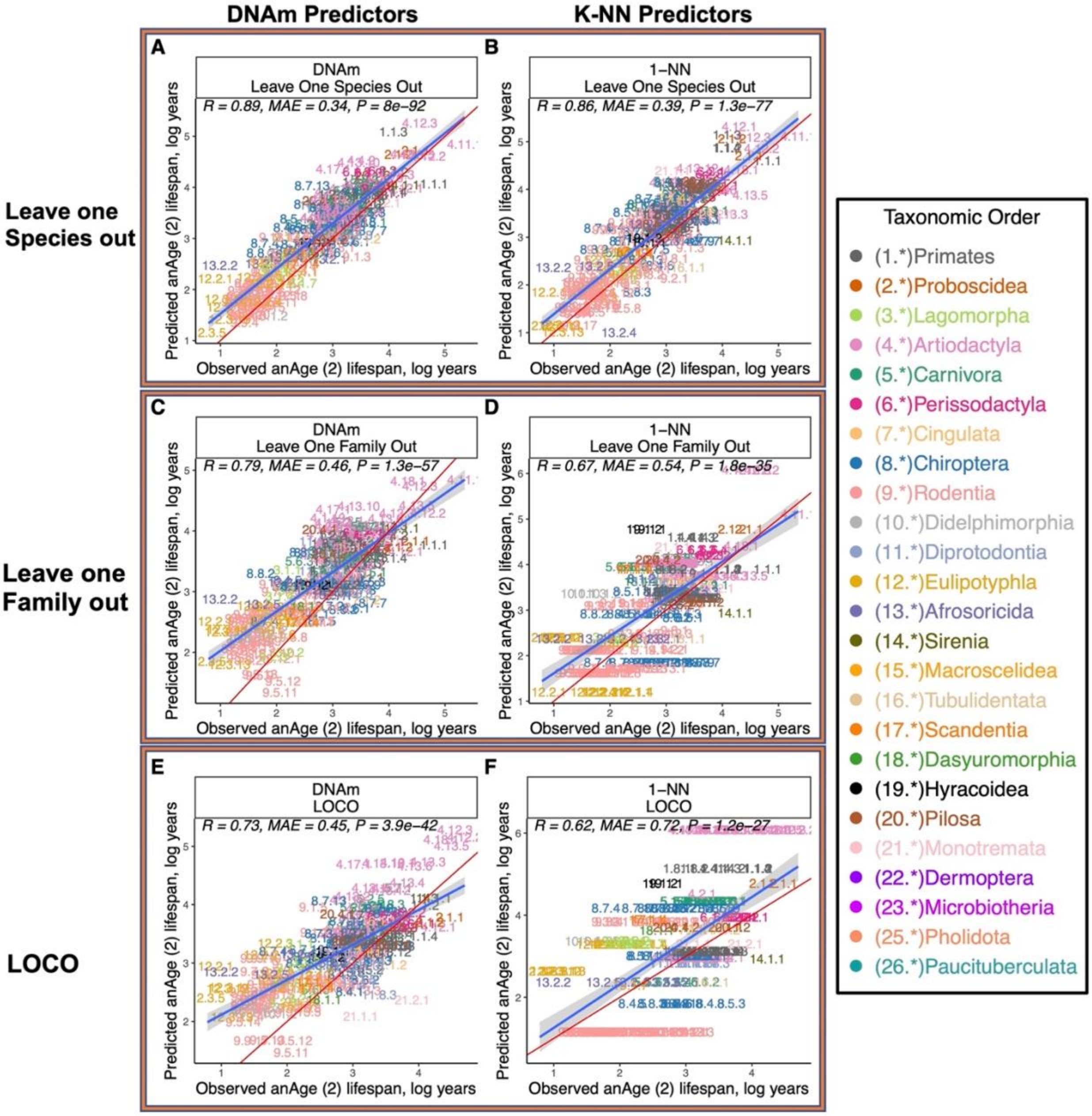

Fig. S12 | Overall comparisons between DNAm lifespan predictors and phylogeny-based predictors.

Various training-test validation analyses of predictors of log (base e) transformed estimates of maximum lifespan. We compared prediction performance between DNAm elastic net predictors and 1-Nearest-Neighbor predictor (k-NN). 1-Nearest-Neighbor predictor utilizes distances from the Mammalian phylogenetic TimeTree (*55*). Results under different training-test separation methods are shown in panels **A**, **B**, DNAm and k-NN predictors test set predictions under leave-one-species-out (LOSO) training-test separation scheme; **C**, **D**, DNAm and k-NN predictors test set predictions under leave-one-family-out training-test separation; **E**, **F**, DNAm and k-NN predictors test set predictions under leave-one-clade-out (LOCO) training-test separation. LOCO (leave-one-clade-out) is defined as, for orders with more than 20 species (Rodentia, Artiodactyla,

Chiroptera, Primates, Carnivora, and Eulipotyphla), leaving out all member species except the longest-living and shortest-living species. MAE abbreviates median absolute errors from the regression errors; r and p denote Pearson's correlation and p-values, respectively. Numbers and colors are the mammalian species number and order annotation consistent with those of other Figures. Numeric values can be found in **table S1**. Shaded areas represent 95% confidence intervals of the simple linear regression line.

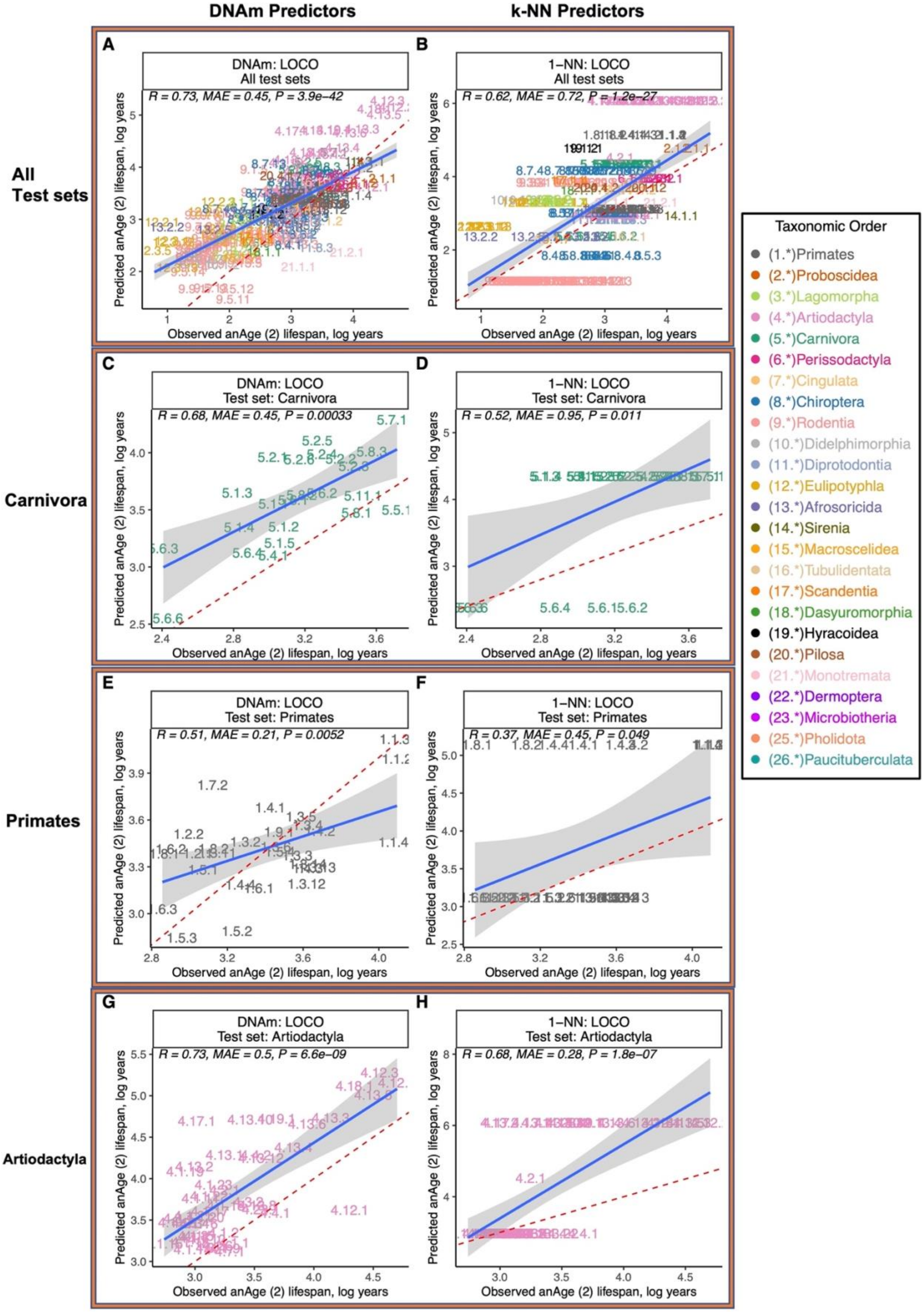

DNAm Predictors
k-NN Predictors
All Test sets
Carnivora
Primates
Artiodactyla
A
DNAm: LOCO
All test sets
R = 0.73, MAE = 0.45, P = 3.9e−42
B
1−NN: LOCO
All test sets
R = 0.62, MAE = 0.72, P = 1.2e−27
C
DNAm: LOCO
Test set: Carnivora
R = 0.68, MAE = 0.45, P = 0.00033
D
1−NN: LOCO
Test set: Carnivora
R = 0.52, MAE = 0.95, P = 0.011
E
DNAm: LOCO
Test set: Primates
R = 0.51, MAE = 0.21, P = 0.0052
F
1−NN: LOCO
Test set: Primates
R = 0.37, MAE = 0.45, P = 0.049
G
DNAm: LOCO
Test set: Artiodactyla
R = 0.73, MAE = 0.5, P = 6.6e−09
H
1−NN: LOCO
Test set: Artiodactyla
R = 0.68, MAE = 0.28, P = 1.8e−07
Predicted anAge (2) lifespan, log years
Observed anAge (2) lifespan, log years
Taxonomic Order
(1.*)Primates
(2.*)Proboscidea
(3.*)Lagomorpha
(4.*)Artiodactyla
(5.*)Carnivora
(6.*)Perissodactyla
(7.*)Cingulata
(8.*)Chiroptera
(9.*)Rodentia
(10.*)Didelphimorphia
(11.*)Diprotodontia
(12.*)Eulipotyphla
(13.*)Afrosoricida
(14.*)Sirenia
(15.*)Macroscelidea
(16.*)Tubulidentata
(17.*)Scandentia
(18.*)Dasyuromorphia
(19.*)Hyracoidea
(20.*)Pilosa
(21.*)Monotremata
(22.*)Dermoptera
(23.*)Microbiotheria
(25.*)Pholidota
(26.*)Paucituberculata

Fig. S13 | Taxonomic order breakdown of DNAm lifespan predictors and Phylogeny-based Predictors under LOCO.

A breakdown of predictor performance in large taxonomic orders under LOCO. We compared prediction performance between DNAm elastic net predictors and 1-Nearest-Neighbor predictor (k-NN). 1-Nearest-Neighbor predictor utilizes distances from the Mammalian phylogenetic TimeTree (*55*). **A**, DNAm predictor's test set predictions leave-one-clade-out (LOCO) training-test separation scheme; **B**, k-NN predictor's test set predictions under LOCO; **C**, **D**, DNAm and k-NN predictors, respectively, test set predictions of lifespan for all species belonging to Carnivora under LOCO; **E**, **F**, DNAm and k-NN predictors, respectively, test set predictions of lifespan for all species belonging to Primates under LOCO; **G**, **H** DNAm and k-NN predictors, respectively, test set predictions of lifespan for all species belonging to Artiodactyla under LOCO. MAE abbreviates median absolute errors from the regression errors; r and p denote Pearson's correlation and p-values, respectively. Numbers and colors are the mammalian species number and order annotation consistent with those of fig. S1. Numeric values can be found in **table S1**. Shaded areas represent 95% confidence intervals of the simple linear regression line. Panels **A** and **B** are analogous to those of **Fig. 2C,D**.

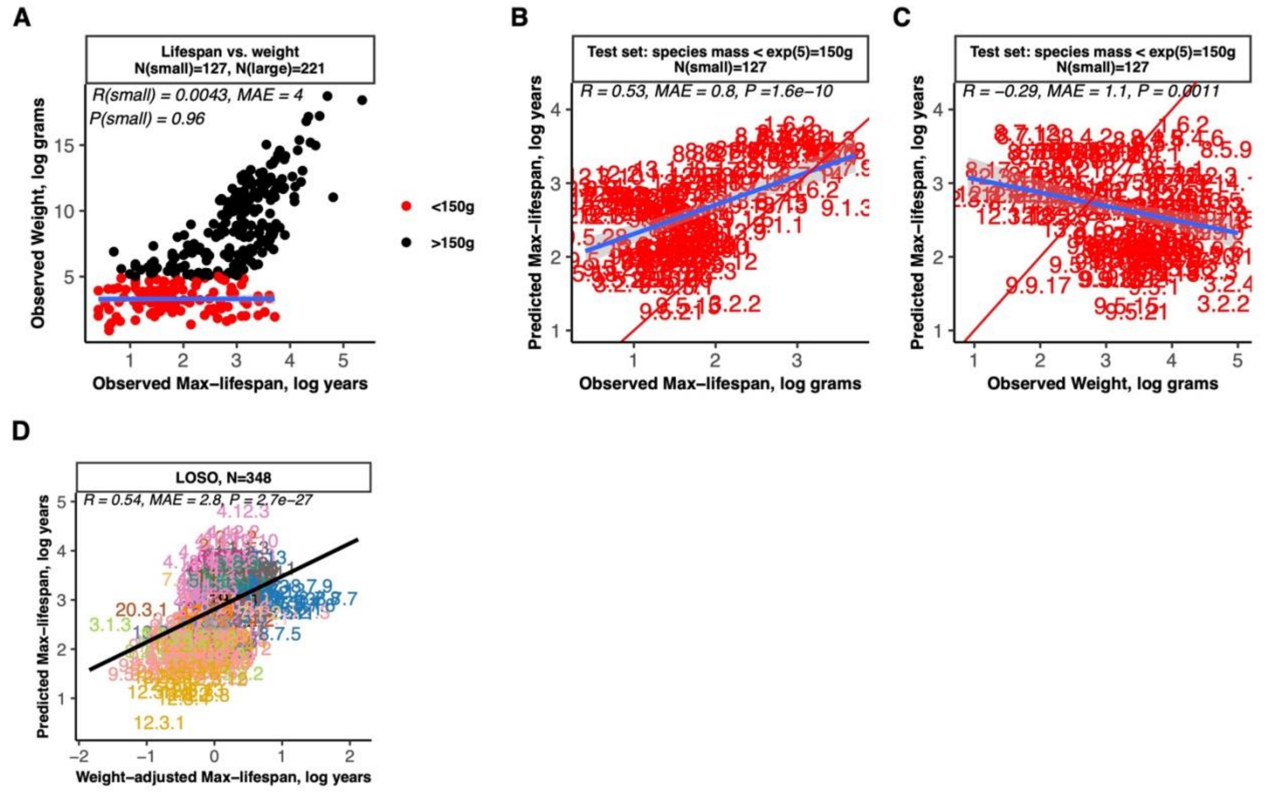

Fig. S14 | DNAm lifespan predictions do not reflect confounding by adult weight.

a-c, Report results for a DNAm max lifespan predictor trained on mammal species with an average weight under 150 grams (small mammals). Panels **A**, observed (log) adult body weight vs. observed (log) maximum lifespan in all mammalian species within the data set, color-coded by small-size indicator (more than 150 grams); **B**, test set predictions for the maximum lifespan in small-sized (<150 grams) mammalian species vs. observed (log) maximum lifespan; **C**, test set predictions for the maximum lifespan in small-sized (<150 grams) mammalian species vs. observed (log) adult body weight. MAE abbreviates median absolute errors from the regression errors; r and p denote Pearson's correlation and p-values, respectively. Numbers are the mammalian species number annotation consistent with those of other Figures. Numeric values can be found in **table S1**. Shaded areas represent 95% confidence intervals of the simple linear regression line. **D**, Results for the final version of the tissue-agnostic DNAm predictor of maximum lifespan. Predicted maximum lifespan (on the log scale, y-axis) versus the corresponding adult weight adjusted version (x-axis). Specifically, the weight adjusted version of log maximum lifespan was defined as raw residual resulting from regressing log transformed predicted maximum lifespan on the log transformed average adult weight of the species.

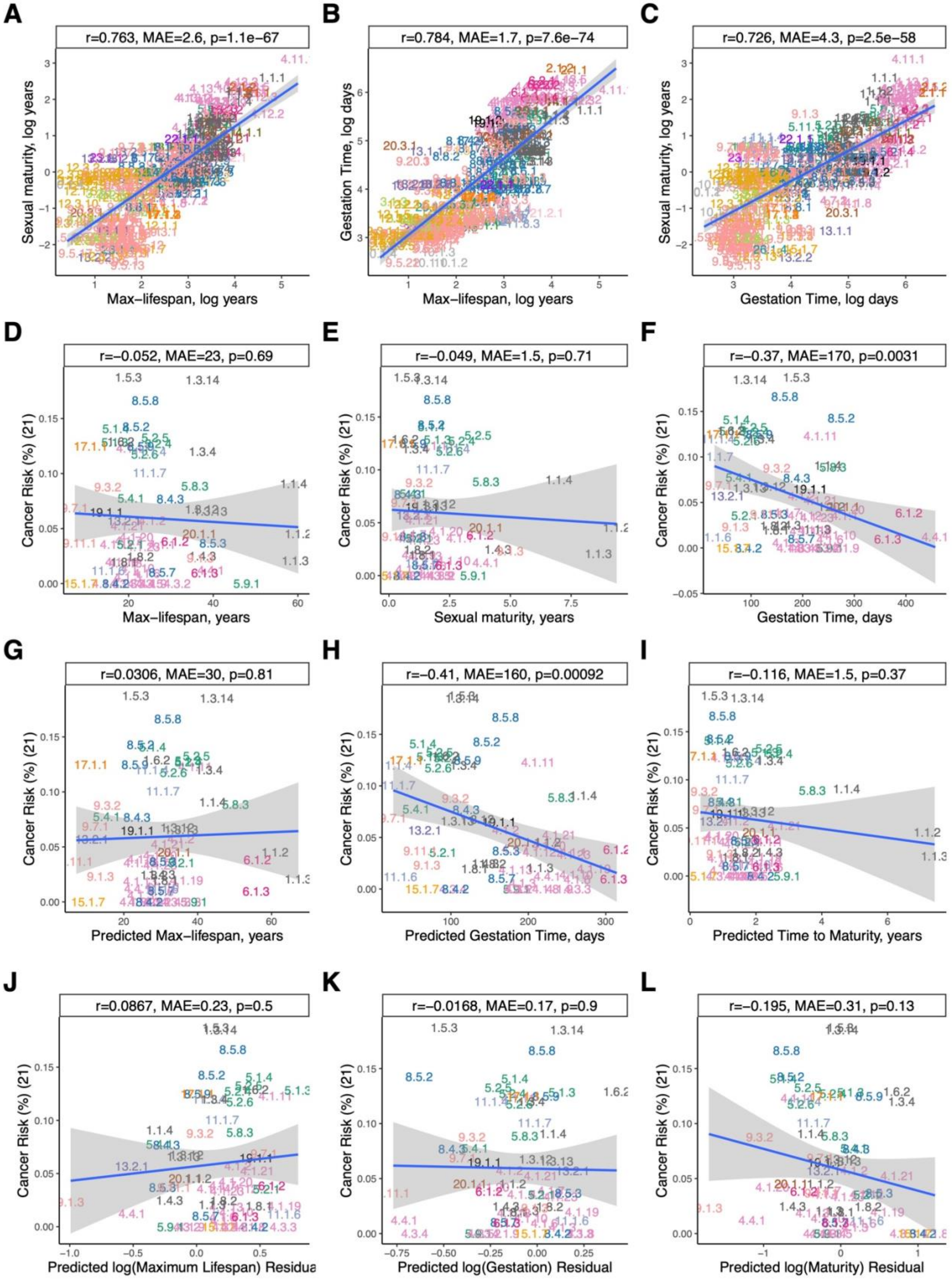
A
r=0.763, MAE=2.6, p=1.1e−67
Sexual maturity, log years
Max−lifespan, log years
B
r=0.784, MAE=1.7, p=7.6e−74
Gestation Time, log days
Max−lifespan, log years
C
r=0.726, MAE=4.3, p=2.5e−58
Sexual maturity, log years
Gestation Time, log days
D
r=−0.052, MAE=23, p=0.69
Cancer Risk (%) (21)
Max−lifespan, years
E
r=−0.049, MAE=1.5, p=0.71
Cancer Risk (%) (21)
Sexual maturity, years
F
r=−0.37, MAE=170, p=0.0031
Cancer Risk (%) (21)
Gestation Time, days
G
r=0.0306, MAE=30, p=0.81
Cancer Risk (%) (21)
Predicted Max−lifespan, years
H
r=−0.41, MAE=160, p=0.00092
Cancer Risk (%) (21)
Predicted Gestation Time, days
I
r=−0.116, MAE=1.5, p=0.37
Cancer Risk (%) (21)
Predicted Time to Maturity, years
J
r=0.0867, MAE=0.23, p=0.5
Cancer Risk (%) (21)
Predicted log(Maximum Lifespan) Residua
K
r=−0.0168, MAE=0.17, p=0.9
Cancer Risk (%) (21)
Predicted log(Gestation) Residual
L
r=−0.195, MAE=0.31, p=0.13
Cancer Risk (%) (21)
Predicted log(Maturity) Residual

Fig. S15 | Relationships between observed and epigenetic estimates of mammalian life history traits, including mammalian cancer risk.

A-F, Panels depict log-transformed relationships between observed variables: **A**. Age at sexual maturity and maximum lifespan **B**. Gestation time and maximum lifespan **C**. Sexual maturity time and gestation time **D**. Cancer risk and maximum lifespan **E**. Cancer risk and sexual maturity **F.** cancer risk and gestation time. **G-I**, estimates of mammalian cancer risk (*21*, y-axis) are plotted against their corresponding epigenetic estimates: **G.** Maximum lifespan, **H**. Gestation time, **I**. age at sexual maturity. **J-L**, this set is analogous to **G-I**, but the x-axis reports residuals derived from regressing the epigenetic estimate of the life history trait on its observed value (on the log scale): **J**. Log maximum lifespan, **K**. Log gestation time, **L**. Log-transformed age at sexual maturity. "MAE" represents median absolute errors from the regression errors, while "r" and "p" signify Pearson's correlation and p-values, respectively. Numbering and colors correspond to the mammalian species number and order, consistent with those in **Fig. 1**. Shaded areas illustrate the 95% confidence intervals of the simple linear regression line. log denotes the natural logarithm, i.e., base e.

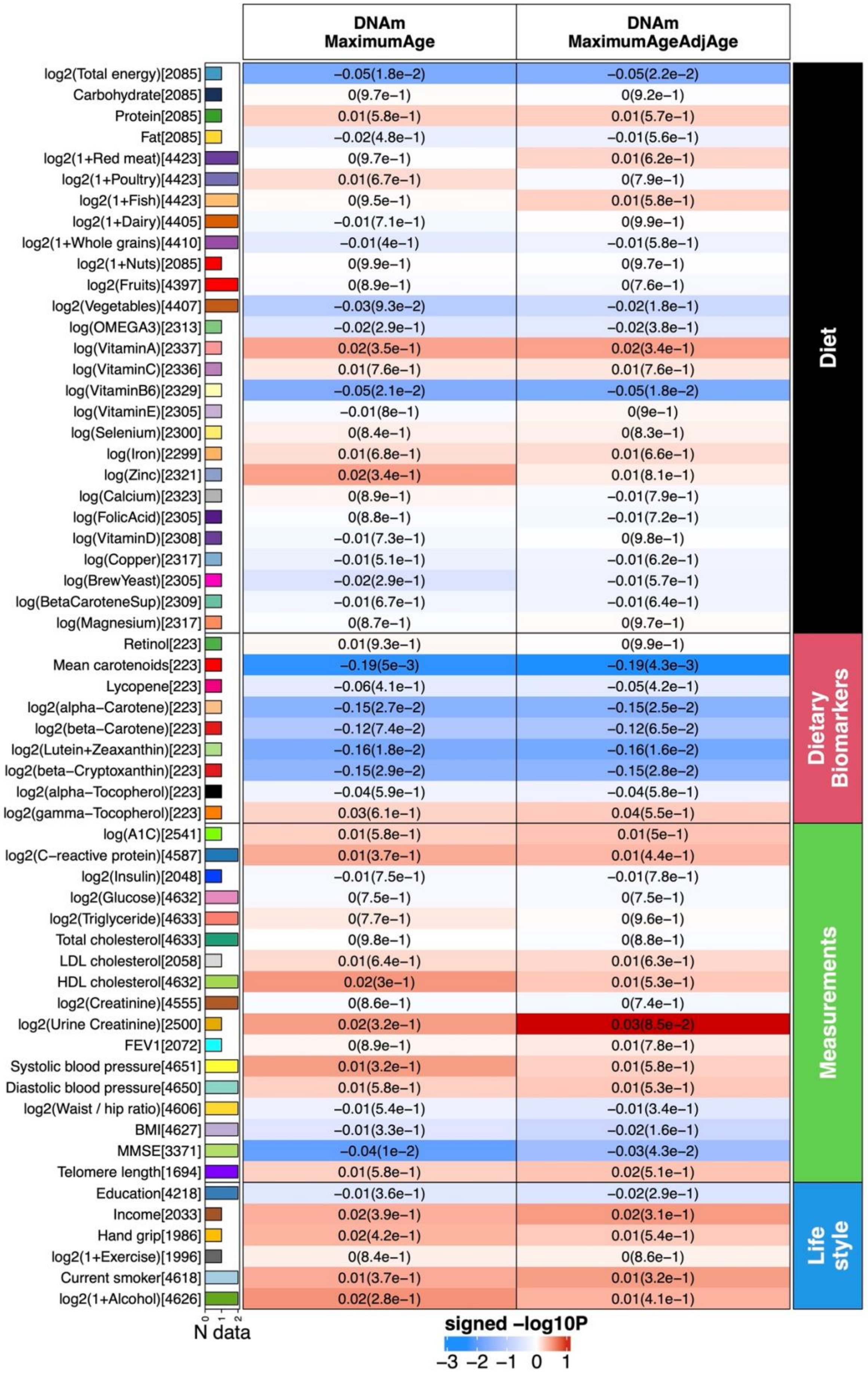
DNAm MaximumAge
DNAm MaximumAgeAdjAge
log2(Total energy)[2085] −0.05(1.8e−2) −0.05(2.2e−2)
Carbohydrate[2085] 0(9.7e−1) 0(9.2e−1)
Protein[2085] 0.01(5.8e−1) 0.01(5.7e−1)
Fat[2085] −0.02(4.8e−1) −0.01(5.6e−1)
log2(1+Red meat)[4423] 0(9.7e−1) 0.01(6.2e−1)
log2(1+Poultry)[4423] 0.01(6.7e−1) 0(7.9e−1)
log2(1+Fish)[4423] 0(9.5e−1) 0.01(5.8e−1)
log2(1+Dairy)[4405] −0.01(7.1e−1) 0(9.9e−1)
log2(1+Whole grains)[4410] −0.01(4e−1) −0.01(5.8e−1)
log2(1+Nuts)[2085] 0(9.9e−1) 0(9.7e−1)
log2(Fruits)[4397] 0(8.9e−1) 0(7.6e−1)
log2(Vegetables)[4407] −0.03(9.3e−2) −0.02(1.8e−1)
log(OMEGA3)[2313] −0.02(2.9e−1) −0.02(3.8e−1)
log(VitaminA)[2337] 0.02(3.5e−1) 0.02(3.4e−1)
log(VitaminC)[2336] 0.01(7.6e−1) 0.01(7.6e−1)
log(VitaminB6)[2329] −0.05(2.1e−2) −0.05(1.8e−2)
log(VitaminE)[2305] −0.01(8e−1) 0(9e−1)
log(Selenium)[2300] 0(8.4e−1) 0(8.3e−1)
log(Iron)[2299] 0.01(6.8e−1) 0.01(6.6e−1)
log(Zinc)[2321] 0.02(3.4e−1) 0.01(8.1e−1)
log(Calcium)[2323] 0(8.9e−1) −0.01(7.9e−1)
log(FolicAcid)[2305] 0(8.8e−1) −0.01(7.2e−1)
log(VitaminD)[2308] −0.01(7.3e−1) 0(9.8e−1)
log(Copper)[2317] −0.01(5.1e−1) −0.01(6.2e−1)
log(BrewYeast)[2305] −0.02(2.9e−1) −0.01(5.7e−1)
log(BetaCaroteneSup)[2309] −0.01(6.7e−1) −0.01(6.4e−1)
log(Magnesium)[2317] 0(8.7e−1) 0(9.7e−1)
Retinol[223] 0.01(9.3e−1) 0(9.9e−1)
Mean carotenoids[223] −0.19(5e−3) −0.19(4.3e−3)
Lycopene[223] −0.06(4.1e−1) −0.05(4.2e−1)
log2(alpha−Carotene)[223] −0.15(2.7e−2) −0.15(2.5e−2)
log2(beta−Carotene)[223] −0.12(7.4e−2) −0.12(6.5e−2)
log2(Lutein+Zeaxanthin)[223] −0.16(1.8e−2) −0.16(1.6e−2)
log2(beta−Cryptoxanthin)[223] −0.15(2.9e−2) −0.15(2.8e−2)
log2(alpha−Tocopherol)[223] −0.04(5.9e−1) −0.04(5.8e−1)
log2(gamma−Tocopherol)[223] 0.03(6.1e−1) 0.04(5.5e−1)
log(A1C)[2541] 0.01(5.8e−1) 0.01(5e−1)
log2(C−reactive protein)[4587] 0.01(3.7e−1) 0.01(4.4e−1)
log2(Insulin)[2048] −0.01(7.5e−1) −0.01(7.8e−1)
log2(Glucose)[4632] 0(7.5e−1) 0(7.5e−1)
log2(Triglyceride)[4633] 0(7.7e−1) 0(9.6e−1)
Total cholesterol[4633] 0(9.8e−1) 0(8.8e−1)
LDL cholesterol[2058] 0.01(6.4e−1) 0.01(6.3e−1)
HDL cholesterol[4632] 0.02(3e−1) 0.01(5.3e−1)
log2(Creatinine)[4555] 0(8.6e−1) 0(7.4e−1)
log2(Urine Creatinine)[2500] 0.02(3.2e−1) 0.03(8.5e−2)
FEV1[2072] 0(8.9e−1) 0.01(7.8e−1)
Systolic blood pressure[4651] 0.01(3.2e−1) 0.01(5.8e−1)
Diastolic blood pressure[4650] 0.01(5.8e−1) 0.01(5.3e−1)
log2(Waist / hip ratio)[4606] −0.01(5.4e−1) −0.01(3.4e−1)
BMI[4627] −0.01(3.3e−1) −0.02(1.6e−1)
MMSE[3371] −0.04(1e−2) −0.03(4.3e−2)
Telomere length[1694] 0.01(5.8e−1) 0.02(5.1e−1)
Education[4218] −0.01(3.6e−1) −0.02(2.9e−1)
Income[2033] 0.02(3.9e−1) 0.02(3.1e−1)
Hand grip[1986] 0.02(4.2e−1) 0.01(5.4e−1)
log2(1+Exercise)[1996] 0(8.4e−1) 0(8.6e−1)
Current smoker[4618] 0.01(3.7e−1) 0.01(3.2e−1)
log2(1+Alcohol)[4626] 0.02(2.8e−1) 0.01(4.1e−1)
Diet
Dietary Biomarkers
Measurements
Life style
0 1 2
N data
signed −log10P
−3 −2 −1 0 1

Fig. S16 | Human epidemiological cohort studies of diet and clinical biomarkers.

We performed a correlation analysis between (1) our methylation-based estimator of maximum lifespan (first column) and its age adjusted version (second column) and (2) 59 variables spanning diet, clinically relevant measurements, and lifestyle factors. Comprehensive details of these variables can be found in reference (*57*). We conducted a robust correlation analysis (biweight midcorrelation, bicor) between (1) our methylation based measures (columns), and (2) 59 variables encompassing 27 self-reported dietary factors, 9 dietary biomarkers, 17 clinical measurements related to vital signs, metabolic traits, inflammatory markers, cognitive and lung function, central adiposity, leukocyte telomere length, and 6 lifestyle factors. This bicor analysis was applied to individuals from both the Framingham Heart Study (up to n=2544) and Women's Health Initiative (up to n=2107), stratified by gender and ethnic category within each respective cohort. The results were consolidated using fixed-effects meta-analysis models, weighted by inverse variance, generating a meta-estimate of bicor and meta P-value. The clinical biomarkers in FHS offspring cohort were measured during the 8th examination aligned with the measures of DNA methylation profiles. The 9 dietary biomarkers, however, were only available in the WHI cohort, with measurements taken from fasting plasma collected at baseline. Food groups and nutrients considered were comprehensive, encompassing all types and preparation methods; for instance, folic acid included both synthetic and natural forms, and dairy encompassed cheese and all varieties of milk. Further details on the individual diet variables of the WHI cohort can be found in our previous study (*57*).

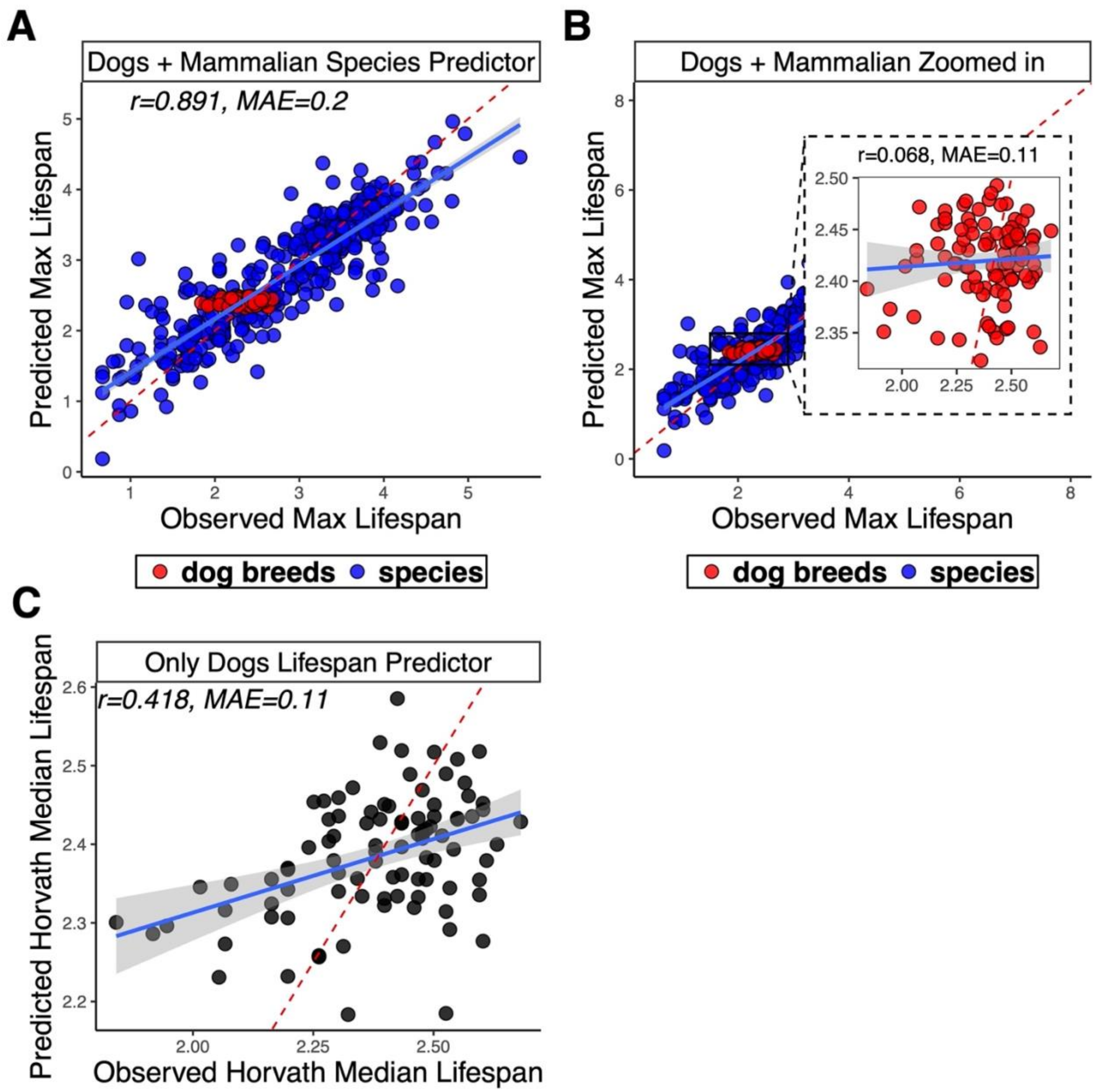

Fig. S17 | Maximum Mammalian Lifespan Predictor which discriminates dog breeds.

The y-axis in each panel represents the cross-validation estimates of the log-transformed maximum lifespan, while the x-axis displays the observed values from AnAge or the canine study (*41*). **a**, a multivariate predictor of maximum lifespan trained across all mammalian species, including 93 dog breeds. This predictor was constructed similarly to that in **Fig. 1**, with a key difference: instead of using a single data point for the species Canis lupus familiaris, we differentiated into 93 separate entries for various dog breeds. The lifespan data for these breeds and their corresponding methylation data are detailed in reference (*41*).

B, This is a detailed view of panel **A**, focusing solely on dog breeds. The negligible Pearson correlation (r=0.068) reflected the predictor's ineffectiveness in accurately distinguishing between longer- and shorter-lived dog breeds. **C**, a different multivariate predictor is trained exclusively on the dog breed data. The multivariate predictor for the (log-transformed) median lifespan of dog breeds shows a modest correlation (r=0.42).

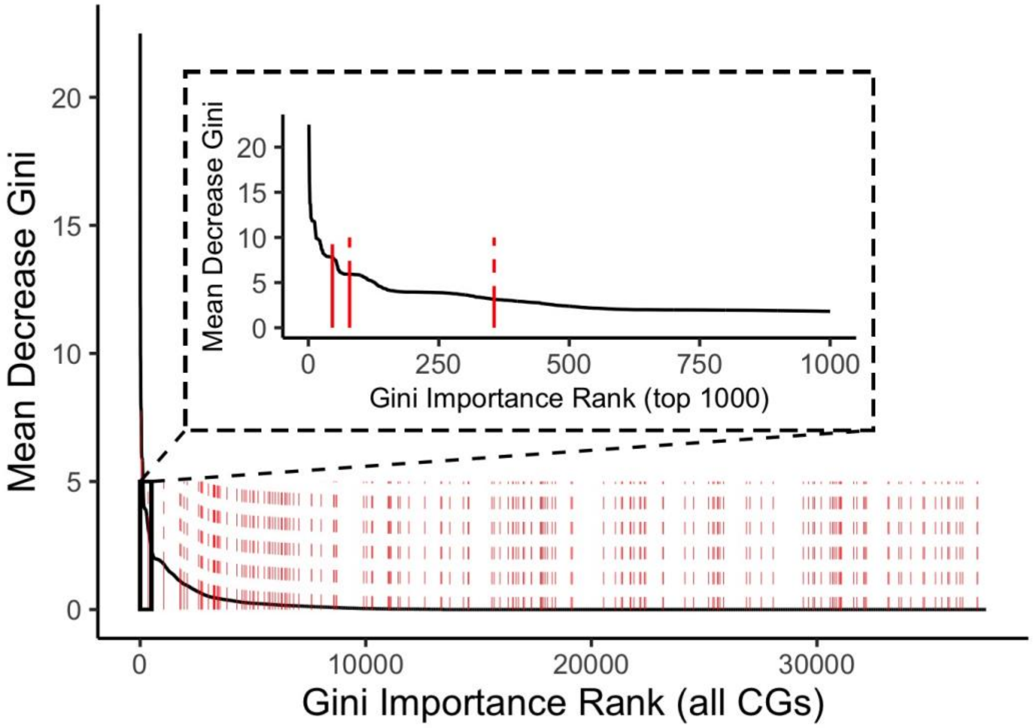

Fig. S18 | Species-predictive CpGs vs. linear lifespan predictor CpGs

It is of interest to characterize which CpGs are most important for predicting species according to our random forest predictor (**table 1**). Towards this end, we used the Gini index based measure of variable importance from our trained Random Forest species classifier to rank CpGs according to their importance for predicting species. In this figure, the line plot corresponds to all 37492 CpGs used by the Random Forest species classifier, ranked by mean reduction in Gini index, from high to low. The CpGs are ordered by mean reduction in Gini index along the x-axis, indicating their rankings. In addition, the figure overlays the 152 CpGs selected by the linear lifespan predictor, annotated in red lines, among species Random Forest's Gini index ranking. We observe lifespan CpGs evenly spread-out throughout the Gini ranking. We highlight the top 1000 Random Forest CGs that are the overwhelmingly dominant Random Forest features, of which only 3 out of the 152 lifespan predictor CpGs are members. Majority of the lifespan predictor CpGs resulted in close to no Gini index reduction when removed from the random forest, implying them being minimally "influential" in classifying Mammalian species.

Supplementary Data Excel File Captions

Table S1 Mammalian Species Information

Table listing all Mammalian species used for this article. In addition, mammalian taxonomic Orders, designated Mammalian numbers, common names, observed maximum lifespan, sample sizes, time to sexual maturity, body mass, and tissues collected are provided for each species as well.

Table S2 Data Description

Taxonomic Orders information is summarized, including number of species per Order, total sample sizes per Order, and longest living species per Order.

Table S3 Lifespan Predictions by Tissues - Final Model

This table summarizes final model predictions on individual sample predictions aggregated by species-tissue strata. The final model* is trained on all available species-averaged data as the training set, building a final predictor. The predictors are trained on log-scale. Columns reporting predictions in units of years (non-log) are taken the exponential of the log predictions, and will therefore exhibit a non-linear log-shaped relations; Member mean*, prediction model trained on species-averaged data same as above, but fitted to individual members of each species, and subsequently mean predictions for each species are reported. Sample sizes reported here do not include all of the hybrid animals, as well as those whose species information remains unknown. Male - Female Ratio*, using female lifespan predictions as denominator, find the ratio of male predicted lifespan / female predicted lifespan.

Table S4 DNAm Maximum Lifespan Predictions by Species

This table summarizes final model predictions on individual sample predictions aggregated by species strata. It also includes species predictions LOSO* cross-validation, Leave-one-species-out test set prediction estimates, where test sets are separated as each species, and predictions are based on all other species (See Main **Fig. 1**). Final Model*, in addition to the LOSO model, we fit the model with same specifications on all available data. All predictors are train on log-scale to counter data skewness. Columns reporting predictions in units of years (non-log) are taken the exponential of the log predictions, and will therefore exhibit a non-linear log-shaped relations; Member median*, prediction model trained on species-averaged data same as above, but fitted to individual members of each species, and subsequent median predictions for each species are reported. Sample sizes reported here do not inlucde all of the hybrid animals, as well as those whose species information remains unknown. In Column, “Female – Male Significant Tissues,” a “+” sign denotes Female minus male mean predicted DNAm lifespan is positive with an unadjusted p-value =< 0.05, “-” vice versa, and “.” denotes a p-value > 0.05

Table S5 Tissue-agnostic Final Maximum Lifespan (log-years) Prediction Model

Coefficients

This table reports the final tissue-agnostic maximum lifespan (log scale) prediction model coefficients. This final regularized regression model, Elastic net, was trained on 348 species-level

summary data, in which each summary CpG value is a species-wide average CpG methylation measurement. However, the final model can be fitted to either individual or aggregated samples.

Table S6 Tissue-agnostic Final Gestation Time (log-days) Prediction Model Coefficients

This table reports the final tissue-agnostic gestation time (log scale) prediction model coefficients. This final regularized regression model, Elastic net, was trained on 348 species-level summary data, in which each summary CpG value is a species-wide average CpG methylation measurement. However, the final model can be fitted to either individual or aggregated samples.

Table S7 Tissue-agnostic Final Time-to-Sexual-Maturity (log-years) Prediction Model Coefficients

This table reports the final tissue-agnostic time to sexual maturity (log scale) prediction model coefficients. This final regularized regression model, Elastic net, was trained on 348 species-level summary data, in which each summary CpG value is a species-wide average CpG methylation measurement. However, the final model can be fitted to either individual or aggregated samples.

Table S8 Tissue-aware Final Maximum Lifespan (log-years) Prediction Model Coefficients

This table reports the final tissue-aware maximum lifespan (log scale) prediction model coefficients. This final regularized regression model, Elastic net, was trained on 348 species-level summary data, in which each summary CpG value is a species-wide average CpG methylation measurement. However, the final model can be fitted to either individual or aggregated samples.

Table S9 Tissue-aware Final Gestation Time (log-days) Prediction Model Coefficients

This table reports the final tissue-aware gestation time (log scale) prediction model coefficients. This final regularized regression model, Elastic net, was trained on 348 species-level summary data, in which each summary CpG value is a species-wide average CpG methylation measurement. However, the final model can be fitted to either individual or aggregated samples.

Table S10 Tissue-aware Final Time-to-Sexual-Maturity (log-years) Prediction Model Coefficients

This table reports the final tissue-aware time to sexual maturity (log scale) prediction model coefficients. This final regularized regression model, Elastic net, was trained on 348 species-level summary data, in which each summary CpG value is a species-wide average CpG methylation measurement. However, the final model can be fitted to either individual or aggregated samples.

Table S11 Museum of Vertebrates, UC Berkeley Catalog Numbers

This table reports all samples used in this article that come from Museum of Vertebrates, UC Berkeley, with their corresponding Catalog Numbers, species Latin name, species common name, and tissue type if applicable.

REFERENCES AND NOTES

1. J. C. George, J. Bada, J. Zeh, L. Scott, S. E. Brown, T. O'Hara, R. Suydam, Age and growth estimates of bowhead whales (Balaena mysticetus) via aspartic acid racemization. *Can. J. Zool.* **77**, 571–580 (1999).

2. J. P. de Magalhaes, J. Costa, G. M. Church, An analysis of the relationship between metabolism, developmental schedules, and longevity using phylogenetic independent contrasts. *J. Gerontol. A Biol. Sci. Med. Sci.* **62**, 149–160 (2007).

3. S. N. Austad, Methusaleh's Zoo: How nature provides us with clues for extending human health span. *J. Comp. Pathol.* **142**, S10–S21 (2010).

4. V. Gorbunova, A. Seluanov, Coevolution of telomerase activity and body mass in mammals: From mice to beavers. *Mech. Ageing Dev.* **130**, 3–9 (2009).

5. J. M. Harper, A. B. Salmon, S. F. Leiser, A. T. Galecki, R. A. Miller, Skin-derived fibroblasts from long-lived species are resistant to some, but not all, lethal stresses and to the mitochondrial inhibitor rotenone. *Aging Cell* **6**, 1–13 (2007).

6. X. Tian, D. Firsanov, Z. Zhang, Y. Cheng, L. Luo, G. Tombline, R. Tan, M. Simon, S. Henderson, J. Steffan, A. Goldfarb, J. Tam, K. Zheng, A. Cornwell, A. Johnson, J. N. Yang, Z. Mao, B. Manta, W. Dang, Z. Zhang, J. Vijg, A. Wolfe, K. Moody, B. K. Kennedy, D. Bohmann, V. N. Gladyshev, A. Seluanov, V. Gorbunova, SIRT6 is responsible for more efficient DNA double-strand break repair in long-lived species. *Cell* **177**, 622–638.e22 (2019).

7. T. A. Rando, H. Y. Chang, Aging, rejuvenation, and epigenetic reprogramming: Resetting the aging clock. *Cell* **148**, 46–57 (2012).

8. J. P. de Magalhaes, Programmatic features of aging originating in development: Aging mechanisms beyond molecular damage? *FASEB J.* **26**, 4821–4826 (2012).

9. J. Mitteldorf, An epigenetic clock controls aging. *Biogerontology* **17**, 257–265 (2016).

10. B. Mayne, O. Berry, C. Davies, J. Farley, S. Jarman, A genomic predictor of lifespan in vertebrates. *Sci. Rep.* **9**, 17866 (2019).

11. J.-H. Yang, P. T. Griffin, D. L. Vera, J. K. Apostolides, M. Hayano, M. V. Meer, E. L. Salfati, Q. Su, E. M. Munding, M. Blanchette, M. Bhakta, Z. Dou, C. Xu, J. W. Pippin, M. L. Creswell, B. L. O'Connell, R. E. Green, B. A. Garcia, S. L. Berger, P. Oberdoerffer, S. J. Shankland, V. N. Gladyshev, L. A. Rajman, A. R. Pfenning, D. A. Sinclair, Erosion of the epigenetic landscape and loss of cellular identity as a cause of aging in mammals. *SSRN Electron. J.* **186**, 305–326.e27 (2019).

12. R. Lowe, C. Barton, C. A. Jenkins, C. Ernst, O. Forman, D. S. Fernandez-Twinn, C. Bock, S. J. Rossiter, C. G. Faulkes, S. E. Ozanne, L. Walter, D. T. Odom, C. Mellersh, V. K. Rakyan, Ageing-associated DNA methylation dynamics are a molecular readout of lifespan variation among mammalian species. *Genome Biol.* **19**, 22 (2018).

13. P. Sen, P. P. Shah, R. Nativio, S. L. Berger, Epigenetic mechanisms of longevity and aging. *Cell* **166**, 822–839 (2016).

14. L. N. Booth, A. Brunet, The aging epigenome. *Mol. Cell* **62**, 728–744 (2016).

15. G. S. Wilkinson, D. M. Adams, A. Haghani, A. T. Lu, J. Zoller, C. E. Breeze, B. D. Arnold, H. C. Ball, G. Carter, L. N. Cooper, D. K. N. Dechmann, P. Devanna, N. J. Fasel, A. V. Galazyuk, L. Günther, E. Hurme, G. Jones, M. Knörnschild, E. Z. Lattenkamp, C. Z. Li, F. Mayer, J. A. Reinhardt, R. A. Medellin, M. Nagy, B. Pope, M. L. Power, R. D. Ransome, E. C. Teeling, S. C. Vernes, D. Zamora-Mejías, J. Zhang, S. Horvath, DNA methylation predicts age and provides insight into exceptional longevity of bats. *Nat. Commun.* **12**, 1615 (2021).

16. A. Arneson, A. Haghani, M. J. Thompson, M. Pellegrini, S. B. Kwon, H. Vu, E. Maciejewski, M. Yao, C. Z. Li, A. T. Lu, A mammalian methylation array for profiling methylation levels at conserved sequences. *Nat. Commun.* **13**, 1–13 (2022).

17. A. T. Lu, Z. Fei, A. Haghani, T. R. Robeck, J. A. Zoller, C. Z. Li, R. Lowe, Q. Yan, J. Zhang, H. Vu, J. Ablaeva, V. A. Acosta-Rodriguez, D. M. Adams, J. Almunia, A. Aloysius, R. Ardehali, A. Arneson, C. S. Baker, G. Banks, K. Belov, N. C. Bennett, P. Black, D. T. Blumstein, E. K. Bors, C. E. Breeze, R. T. Brooke, J. L. Brown, G. Carter, A. Caulton, J. M. Cavin, L. Chakrabarti, I. Chatzistamou, H. Chen, K. Cheng, P. Chiavellini, O. W. Choi, S. Clarke, L. N. Cooper, M. L. Cossette, J. Day, J. DeYoung, S. DiRocco, C. Dold, E. E. Ehmke, C. K. Emmons, S. Emmrich, E. Erbay, C. Erlacher-Reid, C. G.

Faulkes, S. H. Ferguson, C. J. Finno, J. E. Flower, J. M. Gaillard, E. Garde, L. Gerber, V. N. Gladyshev, V. Gorbunova, R. G. Goya, M. J. Grant, C. B. Green, E. N. Hales, M. B. Hanson, D. W. Hart, M. Haulena, K. Herrick, A. N. Hogan, C. J. Hogg, T. A. Hore, T. Huang, J. C. Izpisua Belmonte, A. J. Jasinska, G. Jones, E. Jourdain, O. Kashpur, H. Katcher, E. Katsumata, V. Kaza, H. Kiaris, M. S. Kobor, P. Kordowitzki, W. R. Koski, M. Kruetzen, S. B. Kwon, B. Larison, S. G. Lee, M. Lehmann, J. F. Lemaitre, A. J. Levine, C. Li, X. Li, A. R. Lim, D. T. S. Lin, D. M. Lindemann, T. J. Little, N. Macoretta, D. Maddox, C. O. Matkin, J. A. Mattison, M. McClure, J. Mergl, J. J. Meudt, G. A. Montano, K. Mozhui, J. Munshi-South, A. Naderi, M. Nagy, P. Narayan, P. W. Nathanielsz, N. B. Nguyen, C. Niehrs, J. K. O'Brien, P. O'Tierney Ginn, D. T. Odom, A. G. Ophir, S. Osborn, E. A. Ostrander, K. M. Parsons, K. C. Paul, M. Pellegrini, K. J. Peters, A. B. Pedersen, J. L. Petersen, D. W. Pietersen, G. M. Pinho, J. Plassais, J. R. Poganik, N. A. Prado, P. Reddy, B. Rey, B. R. Ritz, J. Robbins, M. Rodriguez, J. Russell, E. Rydkina, L. L. Sailer, A. B. Salmon, A. Sanghavi, K. M. Schachtschneider, D. Schmitt, T. Schmitt, L. Schomacher, L. B. Schook, K. E. Sears, A. W. Seifert, A. Seluanov, A. B. A. Shafer, D. Shanmuganayagam, A. V. Shindyapina, M. Simmons, K. Singh, I. Sinha, J. Slone, R. G. Snell, E. Soltanmaohammadi, M. L. Spangler, M. C. Spriggs, L. Staggs, N. Stedman, K. J. Steinman, D. T. Stewart, V. J. Sugrue, B. Szladovits, J. S. Takahashi, M. Takasugi, E. C. Teeling, M. J. Thompson, B. Van Bonn, S. C. Vernes, D. Villar, H. V. Vinters, M. C. Wallingford, N. Wang, R. K. Wayne, G. S. Wilkinson, C. K. Williams, R. W. Williams, X. W. Yang, M. Yao, B. G. Young, B. Zhang, Z. Zhang, P. Zhao, Y. Zhao, W. Zhou, J. Zimmermann, J. Ernst, K. Raj, S. Horvath, Universal DNA methylation age across mammalian tissues. *Nat. Aging* **3**, 1144–1166 (2023).

18. A. Haghani, C. Z. Li, T. R. Robeck, J. Zhang, A. T. Lu, J. Ablaeva, V. A. Acosta-Rodriguez, D. M. Adams, A. N. Alagaili, J. Almunia, A. Aloysius, N. M. S. Amor, R. Ardehali, A. Arneson, C. S. Baker, G. Banks, K. Belov, N. C. Bennett, P. Black, D. T. Blumstein, E. K. Bors, C. E. Breeze, R. T. Brooke, J. L. Brown, G. Carter, A. Caulton, J. M. Cavin, L. Chakrabarti, I. Chatzistamou, A. S. Chavez, H. Chen, K. Cheng, P. Chiavellini, O. W. Choi, S. Clarke, J. A. Cook, L. N. Cooper, M. L. Cossette, J. Day, J. DeYoung, S. Dirocco, C. Dold, J. L. Dunnum, E. E. Ehmke, C. K. Emmons, S. Emmrich, E. Erbay, C. Erlacher-Reid, C. G. Faulkes, Z. Fei, S. H. Ferguson, C. J. Finno, J. E. Flower, J. M. Gaillard, E. Garde, L. Gerber, V. N. Gladyshev, R. G. Goya, M. J. Grant, C. B. Green, M. B. Hanson, D. W. Hart, M. Haulena, K. Herrick, A. N. Hogan, C. J. Hogg, T. A. Hore, T. Huang, J. C. Izpisua Belmonte, A. J. Jasinska, G. Jones, E. Jourdain, O. Kashpur, H. Katcher, E. Katsumata, V. Kaza, H. Kiaris, M. S. Kobor,

P. Kordowitzki, W. R. Koski, M. Krutzen, S. B. Kwon, B. Larison, S. G. Lee, M. Lehmann, J. F. Lemaitre, A. J. Levine, X. Li, C. Li, A. R. Lim, D. T. S. Lin, D. M. Lindemann, S. W. Liphardt, T. J. Little, N. Macoretta, D. Maddox, C. O. Matkin, J. A. Mattison, M. McClure, J. Mergl, J. J. Meudt, G. A. Montano, K. Mozhui, J. Munshi-South, W. J. Murphy, A. Naderi, M. Nagy, P. Narayan, P. W. Nathanielsz, N. B. Nguyen, C. Niehrs, B. Nyamsuren, J. K. O'Brien, P. O. Ginn, D. T. Odom, A. G. Ophir, S. Osborn, E. A. Ostrander, K. M. Parsons, K. C. Paul, A. B. Pedersen, M. Pellegrini, K. J. Peters, J. L. Petersen, D. W. Pietersen, G. M. Pinho, J. Plassais, J. R. Poganik, N. A. Prado, P. Reddy, B. Rey, B. R. Ritz, J. Robbins, M. Rodriguez, J. Russell, E. Rydkina, L. L. Sailer, A. B. Salmon, A. Sanghavi, K. M. Schachtschneider, D. Schmitt, T. Schmitt, L. Schomacher, L. B. Schook, K. E. Sears, A. W. Seifert, A. B. A. Shafer, A. V. Shindyapina, M. Simmons, K. Singh, I. Sinha, J. Slone, R. G. Snell, E. Soltanmohammadi, M. L. Spangler, M. Spriggs, L. Staggs, N. Stedman, K. J. Steinman, D. T. Stewart, V. J. Sugrue, B. Szladovits, J. S. Takahashi, M. Takasugi, E. C. Teeling, M. J. Thompson, B. Van Bonn, S. C. Vernes, D. Villar, H. V. Vinters, H. Vu, M. C. Wallingford, N. Wang, G. S. Wilkinson, R. W. Williams, Q. Yan, M. Yao, B. G. Young, B. Zhang, Z. Zhang, Y. Zhao, P. Zhao, W. Zhou, J. A. Zoller, J. Ernst, A. Seluanov, V. Gorbunova, X. W. Yang, K. Raj, S. Horvath, DNA methylation networks underlying mammalian traits. *Science* **381**, eabq5693 (2023).

19. S. Horvath, J. A. Zoller, A. Haghani, A. T. Lu, K. Raj, A. J. Jasinska, J. A. Mattison, A. B. Salmon, DNA methylation age analysis of rapamycin in common marmosets. *Geroscience* **43**, 2413–2425 (2021).

20. F. Wilcoxon, *Breakthroughs in Statistics: Methodology and Distribution* (Springer, 1992), pp. 196–202.

21. O. Vincze, F. Colchero, J. F. Lemaitre, D. A. Conde, S. Pavard, M. Bieuville, A. O. Urrutia, B. Ujvari, A. M. Boddy, C. C. Maley, F. Thomas, M. Giraudeau, Cancer risk across mammals. *Nature* **601**, 263–267 (2022).

22. R. Basu, Y. Qian, J. J. Kopchick, Mechanisms in endocrinology: Lessons from growth hormone receptor gene-disrupted mice: Are there benefits of endocrine defects? *Eur. J. Endocrinol.* **178**, R155–R181 (2018).

23. H.R. Pilcher. Money for old mice. Competition seeks world's longest-lasting mouse.

Nature News 22, 1-2 (. (2003).

24. L. Fontana, L. Partridge, V. D. Longo, Extending healthy life span—From yeast to humans. *Science* **328**, 321–326 (2010).

25. K. Flurkey, J. Papaconstantinou, R. A. Miller, D. E. Harrison, Lifespan extension and delayed immune and collagen aging in mutant mice with defects in growth hormone production. *Proc. Natl. Acad. Sci. U.S.A.* **98**, 6736–6741 (2001).

26. G. Dominick, D. E. Berryman, E. O. List, J. J. Kopchick, X. Li, R. A. Miller, G. G. Garcia, Regulation of mTOR activity in Snell dwarf and GH receptor gene-disrupted mice. *Endocrinology* **156**, 565–575 (2015).

27. K. T. Coschigano, D. Clemmons, L. L. Bellush, J. J. Kopchick, Assessment of growth parameters and life span of GHR/BP gene-disrupted Mice1. *Endocrinology* **141**, 2608–2613 (2000).

28. E. O. List, D. E. Berryman, K. Funk, A. Jara, B. Kelder, F. Wang, M. B. Stout, X. Zhi, L. Sun, T. A. White, Liver-specific GH receptor gene-disrupted (LiGHRKO) mice have decreased endocrine IGF-I, increased local IGF-I, and altered body size, body composition, and adipokine profiles. *Endocrinology* **155**, 1793–1805 (2014).

29. A. Nagarajan, H. Srivastava, J. Jablonsky, L. Y. Sun, Tissue-Specific GHR Knockout Mice: Tissue-specific GHR knockout mice: An updated review. *Front. Endocrinol.* **11**, 579909 (2020).

30. K. Mozhui, A. T. Lu, C. Z. Li, A. Haghani, J. V. Sandoval-Sierra, Y. Wu, R. W. Williams, S. Horvath, Genetic loci and metabolic states associated with murine epigenetic aging. *eLife* **11**, e75244 (2022).

31. K. Takahashi, S. Yamanaka, Induction of pluripotent stem cells from mouse embryonic and adult fibroblast cultures by defined factors. *Cell* **126**, 663–676 (2006).

32. A. Ocampo, P. Reddy, P. Martinez-Redondo, A. Platero-Luengo, F. Hatanaka, T. Hishida, M. Li, D. Lam, M. Kurita, E. Beyret, T. Araoka, E. Vazquez-Ferrer, D. Donoso, J. L. Roman, J. Xu, C. Rodriguez Esteban, G. Nunez, E. Nunez Delicado, J. M. Campistol, I. Guillen, P. Guillen, J. C. Izpisua Belmonte,

In vivo amelioration of age-associated hallmarks by partial reprogramming. *Cell* **167**, 1719–1733.e1712 (2016).

33. T. J. Sarkar, M. Quarta, S. Mukherjee, A. Colville, P. Paine, L. Doan, C. M. Tran, C. R. Chu, S. Horvath, L. S. Qi, N. Bhutani, T. A. Rando, V. Sebastiano, Transient non-integrative expression of nuclear reprogramming factors promotes multifaceted amelioration of aging in human cells. *Nat. Commun.* **11**, 1545 (2020).

34. M. Abad, L. Mosteiro, C. Pantoja, M. Canamero, T. Rayon, I. Ors, O. Grana, D. Megias, O. Dominguez, D. Martinez, M. Manzanares, S. Ortega, M. Serrano, Reprogramming in vivo produces teratomas and iPS cells with totipotency features. *Nature* **502**, 340–345 (2013).

35. K. C. Browder, P. Reddy, M. Yamamoto, A. Haghani, I. G. Guillen, S. Sahu, C. Wang, Y. Luque, J. Prieto, L. Shi, K. Shojima, T. Hishida, Z. Lai, Q. Li, F. K. Choudhury, W. R. Wong, Y. Liang, D. Sangaraju, W. Sandoval, C. R. Esteban, E. N. Delicado, P. G. Garcia, M. Pawlak, J. A. Vander Heiden, S. Horvath, H. Jasper, J. C. In vivo partial reprogramming alters age-associated molecular changes during physiological aging in mice *Aging* **2**, 243–253 (2022).

36. Y. Lu, B. Brommer, X. Tian, A. Krishnan, M. Meer, C. Wang, D. L. Vera, Q. Zeng, D. Yu, M. S. Bonkowski, J. H. Yang, S. Zhou, E. M. Hoffmann, M. M. Karg, M. B. Schultz, A. E. Kane, N. Davidsohn, E. Korobkina, K. Chwalek, L. A. Rajman, G. M. Church, K. Hochedlinger, V. N. Gladyshev, S. Horvath, M. E. Levine, M. S. Gregory-Ksander, B. R. Ksander, Z. He, D. A. Sinclair, Reprogramming to recover youthful epigenetic information and restore vision. *Nature* **588**, 124–129 (2020).

37. D. Gill, A. Parry, F. Santos, I. Hernando-Herraez, T. M. Stubbs, I. Milagre, W. Reik, Multi-omic rejuvenation of human cells by maturation phase transient reprogramming. *Elife* **11**, e71624 (2022).

38. T. R. Dawber, G. F. Meadors, F. E. Moore, Jr., Epidemiological approaches to heart disease: The Framingham Study. *Am. J. Public Health Nations Health* **41**, 279–286 (1951).

39. G. L. Anderson, J. Manson, R. Wallace, B. Lund, D. Hall, S. Davis, S. Shumaker, C. Y. Wang, E. Stein, R. L. Prentice, Implementation of the Women's Health Initiative study design. *Ann. Epidemiol.* **13**, S5–S17 (2003).

40. Design of the Women's Health Initiative clinical trial and observational study. The Women's Health Initiative Study Group. *Control. Clin. Trials* **19**, 61–109 (1998).

41. S. Horvath, A. T. Lu, A. Haghani, J. A. Zoller, C. Z. Li, A. R. Lim, R. T. Brooke, K. Raj, A. Serres-Armero, D. L. Dreger, A. N. Hogan, J. Plassais, E. A. Ostrander, DNA methylation clocks for dogs and humans. *Proc. Natl. Acad. Sci. U.S.A.* **119**, e2120887119 (2022).

42. J.-F. Lemaître, V. Ronget, M. Tidière, D. Allainé, V. Berger, A. Cohas, F. Colchero, D. A. Conde, M. Garratt, A. Liker, Sex differences in adult lifespan and aging rates of mortality across wild mammals. *Proc. Natl. Acad. Sci. U.S.A.* **117**, 8546–8553 (2020).

43. T. H. Clutton-Brock, K. Isvaran, Sex differences in ageing in natural populations of vertebrates. *Proc. Biol. Sci.* **274**, 3097–3104 (2007).

44. G. A. Marais, J.-M. Gaillard, C. Vieira, I. Plotton, D. Sanlaville, F. Gueyffier, J.-F. Lemaitre, Sex gap in aging and longevity: Can sex chromosomes play a role? *Biol. Sex Differ.* **9**, 1–14 (2018).

45. H. Vu, J. Ernst, Universal annotation of the human genome through integration of over a thousand epigenomic datasets. *Genome Biol.* **23**, 1–37 (2022).

46. W. Zhou, T. J. Triche, Jr., P. W. Laird, H. Shen, SeSAMe: Reducing artifactual detection of DNA methylation by Infinium BeadChips in genomic deletions. *Nucleic Acids Res.* **46**, e123 (2018).

47. J. W. Vaupel, Post-darwinian longevity. *Popul. Dev. Rev.* **29**, 258–269 (2003).

48. Assessing ageing patterns for comparative analyses of mortality curves: Going beyond the use of maximum longevity. *Funct. Ecol.* **34**, 65–75 (2020).

49. A. T. Lu, A. Quach, J. G. Wilson, A. P. Reiner, A. Aviv, K. Raj, L. Hou, A. A. Baccarelli, Y. Li, J. D. Stewart, DNA methylation GrimAge strongly predicts lifespan and healthspan. *Aging* **11**, 303–327 (2019).

50. H. Zou, T. Hastie, Regularization and variable selection via the elastic net. *J. R. Stat. Soc. Ser. B Stat. Methodol.* **67**, 301–320 (2005).

51. Y. Benjamini, Y. Hochberg, Controlling the false discovery rate: A practical and powerful approach to multiple testing. *J. R. Stat. Soc. B Methodol.* **57**, 289–300 (1995).

52. S. M. Ross, *Introduction to Probability and Statistics for Engineers and Scientists* (Academic Press, 2021).

53. A. M. Boddy, L. M. Abegglen, A. P. Pessier, A. Aktipis, J. D. Schiffman, C. C. Maley, C. Witte, Lifetime cancer prevalence and life history traits in mammals. *Evol. Med. Public Health* **2020**, 187–195 (2020).

54. L. M. Abegglen, A. F. Caulin, A. Chan, K. Lee, R. Robinson, M. S. Campbell, W. K. Kiso, D. L. Schmitt, P. J. Waddell, S. Bhaskara, S. T. Jensen, C. C. Maley, J. D. Schiffman, Potential mechanisms for cancer resistance in elephants and comparative cellular response to DNA damage in humans. *JAMA* **314**, 1850–1860 (2015).

55. S. Kumar, G. Stecher, M. Suleski, S. B. Hedges, TimeTree: A resource for timelines, timetrees, and divergence times. *Mol. Biol. Evol.* **34**, 1812–1819 (2017).

56. M. Ohnuki, K. Tanabe, K. Sutou, I. Teramoto, Y. Sawamura, M. Narita, M. Nakamura, Y. Tokunaga, M. Nakamura, A. Watanabe, S. Yamanaka, K. Takahashi, Dynamic regulation of human endogenous retroviruses mediates factor-induced reprogramming and differentiation potential. *Proc. Natl. Acad. Sci. U.S.A.* **111**, 12426–12431 (2014).

57. A. Quach, M. E. Levine, T. Tanaka, A. T. Lu, B. H. Chen, L. Ferrucci, B. Ritz, S. Bandinelli, M. L. Neuhouser, J. M. Beasley, L. Snetselaar, R. B. Wallace, P. S. Tsao, D. Absher, T. L. Assimes, J. D. Stewart, Y. Li, L. Hou, A. A. Baccarelli, E. A. Whitsel, S. Horvath, Epigenetic clock analysis of diet, exercise, education, and lifestyle factors. *Aging* **9**, 419–446 (2017).
